# Rapid Identification of α-Glucosidase Inhibitors from *Phlomis tuberosa* by Sepbox Chromatography and Thin-Layer Chromatography Bioautography

**DOI:** 10.1371/journal.pone.0116922

**Published:** 2015-02-06

**Authors:** Yingbo Yang, Lihua Gu, Ying Xiao, Qing Liu, Haijun Hu, Zhengtao Wang, Kaixian Chen

**Affiliations:** 1 The MOE Key Laboratory of Standardization of Chinese Medicines, Institute of Chinese Materia Medica, Shanghai University of Traditional Chinese Medicine, Shanghai, China; 2 Shanghai R & D Center for Standardization of Chinese Medicines, Shanghai, China; 3 Department of Pharmacy, Changzheng Hospital, Second Military Medical University, Shanghai, China; Johns Hopkins School of Medicine, UNITED STATES

## Abstract

Alpha-glucosidase inhibitors currently form an important basis for developing novel drugs for diabetes treatment. In our preliminary tests, the ethyl acetate fraction of *Phlomis tuberosa* extracts showed significant α-glucosidase inhibitory activity (IC₅₀ = 100 μg/mL). In the present study, a combined method using Sepbox chromatography and thin-layer chromatography (TLC) bioautography was developed to probe α-glucosidase inhibitors further. The ethyl acetate fraction of *P. tuberosa* extracts was separated into 150 individual subfractions within 20 h using Sepbox chromatography. Then, under the guidance of TLC bioautography, 20 compounds were successfully isolated from these fractions, including four new diterpenoids [14-hydroxyabieta-8,11,13-triene-11-carbaldehyde-18-oic-12-carboxy-13-(1-hydroxy-1-methylethyl)-lactone (**1**), 14-hydroxyabieta-8,11,13-triene-17-oic-12-carboxy-13-(1-hydroxy-1-methylethyl)-lactone (**2**), 14,16-dihydroxyabieta-8,11,13-triene-15,17-dioic acid (**3**), and phlomisol (15,16-eposy-8,13(16),14-labdatrien-19-ol) (**4**)], and 16 known compounds. Activity estimation indicated that 15 compounds showed more potent α-glucosidase inhibitory effects (with IC_50_ values in the range 0.067–1.203 mM) than the positive control, acarbose (IC_50_ = 3.72 ± 0.113 mM). This is the first report of separation of α-glucosidase inhibitors from *P. tuberosa*.

## Introduction

Diabetes is one of the most important health issues facing the world in the 21st century [1]. Alpha-glucosidase inhibitors have been used to lower blood glucose for about 20 years now. These compounds show reasonably good efficacy comparable with other oral blood glucose-lowering drugs and, in some parts of the world, are the most commonly prescribed oral diabetes medication, especially in Asian countries [2]. In recent years, investigations of herbal medicines have become increasingly important in the search for new, effective, and safe α-glucosidase inhibitors for diabetes treatment [3].

*Phlomis tuberosa* L. is an Asian folk medicine used as a general roborant for intoxication, tuberculosis, pulmonary and cardiovascular diseases, and rheumatoid arthritis [4]. In preliminary tests in which we screened α-glucosidase inhibitors from medicinal herbs, the ethyl acetate fraction of *P*. *tuberosa* extracts showed significant inhibitory activity against α-glucosidase (IC_50_ = 100 μg/mL). Repeated column chromatography over silica gel is frequently used for purification of bioactive compounds from medicinal herbs. However, this separation method can be a tedious process requiring long time frames and large volumes of organic solvents; irreversible adsorption of samples onto the solid phase, which sometimes results in reductions or disappearance of active compounds, may also occur [5]. Therefore, better purification strategies to assure the efficacy and reliability of α-glucosidase inhibitor identification from *P*. *tuberosa* are necessary.

Automated HPLC/SPE/HPLC coupled separation using a Sepbox system is a standard technology for separating compounds from natural resources; the technique allows automatic processing of samples by up to 30 times faster than conventional processes [6]. An extract can be fractionated into 100–300 substances composed of 1–4 compounds using Sepbox chromatography within less than 30 h [7]. Thin-layer chromatography (TLC) bioautography combines chromatographic separation with *in situ* biological activity determination, allows the direct and rapid localization of active compounds in complex extracts [8]. A TLC bioautographic method has been established to detect α-glucosidase inhibitors in plant extracts [9]. Because of its unique benefit of simultaneous chromatographic fractionation and bioactivity screening, Sepbox chromatography coupled with TLC bioautography is speculated to be an attractive strategy for rapid identification of α-glucosidase inhibitors from *P*. *tuberosa*.

In the present study, Sepbox chromatography and TLC bioautography led to the isolation of four new diterpenoids (**1**, **2**, **3**, **4**), two known diterpenoids (**5**, **6**), three known phenylethanoid glycosides (**7**, **8**, **9**), three known flavonoids (**10**, **11**, **12**), four known iridoids (**13**, **14**, **15**, **16**), and four other compounds (**17**, **18**, **19**, **20**) from *P*. *tuberosa*. The α-glucosidase inhibitory activities of the isolated compounds were subsequently determined.

## Materials and Methods

### General

The automated HPLC/SPE/HPLC experiment was performed using a Sepbox 2D-2000 (Sepiatec, Germany). Silica gel 60 F_254_TLC plates (MN, Germany) were used for TLC analysis. HPLC analysis was performed using an Agilent 1100 HPLC system (Agilent Technologies, Santa Clara, USA) consisting of a G1312A QuatPump, a G1314A DAD detector, a G1313A autosampler, and a G1332A degasser equipped with an Agilent Zorbax SB-C18 column (250 mm × 4.6 mm, i.d. 5 m). Compound purification was performed using a LC-3000 Semi-preparation gradient HPLC system (Beijing Tong Heng Innovation Technology Co., Ltd., China) equipped with a UV-vis detector and a YMC-Pack PRO C18 column (250 mm × 10 mm, i.d. 5 μm; YMC, Japan). Identification of isolated compounds was performed using ^1^H NMR, ^13^C NMR, and MS. NMR spectra were obtained on a Bruker Avance 400 or 600 NMR spectrometer (Bruker Inc., Bremen, Germany). HRESIMS spectra were obtained using a Waters UPLC Premier QTOF spectrometer, and UPLC-MS analysis was performed on an Acquity Waters ultra-performance liquid chromatographic system equipped with a Waters UPLC column (Acquity UPLC BEH C_18_ 1.7 μm, 2.1 mm × 50 mm) and a Micromass ZQ 2000 ESI mass spectrometer. α-d-Glucosidase, 2-naphthyl-α-d-glucopyranoside, Fast Blue B salt, 4-nitrophenyl α-glucopyranoside, and acarbose were purchased from Sigma-Aldrich (St. Louis, MO, USA). All solvents used for chromatography and extraction were of HPLC grade and obtained from Fisher Scientific (Fair Lawn, NJ, USA). All other chemicals used were of analytical grade and applied without further purification.

### Plant material

Roots of *P*. *tuberosa* were collected from Shangdu Town, Inner Mongolia Autonomous Region, China (latitude/longitude, 42°15′11″N/115°59′52″E), in November 2011 and authenticated by Prof. A.O. Wuliji (Inner Mongolia University for the Nationalities). A voucher specimen (No. kgcs-120515) was deposited at Shanghai R&D Center for Standardization of Traditional Chinese Medicines, Shanghai, China.

### Ethics

No specific permissions were required for the described field studies. The locations are neither privately owned nor protected by the Chinese government. No endangered or protected species were sampled.

### Sample preparation

Air-dried, chopped roots (1.0 kg) of *P*. *tuberosa* were extracted thrice using 10 L of 95% ethanol under reflux for 2.5 h. The extracts were combined and evaporated to dryness at 60°C under reduced pressure. The resulting residue was resuspended in distilled water and partitioned thrice in a separatory funnel with an equal volume of ethyl acetate each time. The ethyl acetate layers were combined and then further dried *in vacuo* at ambient temperature for 24 h to produce the ethyl acetate fraction, which was stored at -20°C prior to Sepbox chromatography separation.

### Sepbox chromatography separation

The Sepbox system combines the advantages of HPLC and SPE and was coupled to an HPLC/SPE/HPLC setup to allow two-dimensional separation. In the Sepbox 2D-2000 system used in this study, a crude ethyl acetate fraction of *P*. *tuberosa* (5 g) was absorbed onto C4 reverse-phase resin (4 g) and initially separated into 15 fractions using a C4 reverse phase HPLC column. These fractions were transferred to 15 SPE trap columns. Fractions trapped in each SPE column were passed through a C18 RP HPLC column for subsequent separation; here, elution was performed with solvents different from those used during the first fractionation. Through monitoring of HPLC peaks from UV (254 nm) and ELSD detectors, a total of 150 individual subfractions (named Pt1–150) were collected. The separation conditions are shown in S1 Table.

### TLC analysis

In the present work, a high-performance thin-layer chromatographic system (CAMAG, Switzerland) equipped with an automatic TLC Sampler 4 and a Reprostar 3 with a 12-bit charge-coupled device camera for photo-documentation and controlled by WinCATS-4 software was used. An aliquot of the subfraction solution (20 μL), the ethyl acetate fraction of *P*. *tuberosa* extract (5 mg/mL, 20 μL), acarbose methanol solution (1 mg/mL, 4 μL), or individual pure isolate methanol solution (2.5 mM, 4 μL) was deposited directly (as spots or bands) onto the TLC plates. After migration of the samples with appropriate solvents or no migration, the plates were inspected under ultraviolet light (366 nm) and then subjected to bioautographic assay as described below.

TLC bioautographic assay: TLC bioautographic assay was carried out as described previously in terms of reaction principle and response system [9] but with slight modifications. First, the concentration of α-glucosidase was reduced from 10 U/mL to 2.5 U/mL. Second, the plate was dipped into the reaction solutions (including the enzyme solution, the substrate solution, and the coloration solution), instead of being sprayed with them. Third, the conditions for enzyme incubation were changed from room temperature for 60 min to 37°C for 30 min. Briefly, α-d-glucosidase (750 U) was dissolved in 75 mL of buffer solution (10.25 g of sodium acetate in 250 mL with addition of 0.1 M acetic acid to pH 7.5). The stock solution was kept at -20°C. A stock solution of 25 mL was diluted with the same buffer solution to 100 mL as the enzyme solution, and a substrate solution of 1 mg/mL 2-naphthyl-α-d-glucopyranoside in ethanol was prepared. To probe active spots (or bands) on the TLC plate, the plate was first dipped in the substrate solution, dried under a stream of cold air for complete removal of the solvent, and then dipped into the enzyme solution. For enzyme incubation, the plate was laid flat on plastic plugs in a plastic tank containing a little water, avoiding direct contact between the water and the plate. A cover was placed on the tank to keep the atmosphere humid and incubation was performed at 37°C for 30 min. To detect the active enzyme, the TLC plate was dipped in a solution of Fast Blue B salt (150 mg) in distilled water (100 mL) prepared immediately before use to produce a white spot under a purple background after 5 min.

### HPLC analysis

Subfractions showing α-glucosidase inhibitory activity in bioautographic assay were analyzed by an Agilent 1100 HPLC system, and subfractions determined to contain two or above compounds in HPLC analysis were further purified by Semi-preparative HPLC. The isolated pure compounds (from both direct Sepbox chromatography fractionation and additional Semi-preparative HPLC purification) as well as the ethyl acetate fraction of *P*. *tuberosa* extracts were separately analyzed by an Agilent 1100 HPLC system. The mobile phase was a mixture of water (A) and acetonitrile (B), and the detailed gradient program is given in S2 Table. The flow rate was 1 mL/min, and the effluents were monitored by a DAD detector at 280 nm.

### α-Glucosidase inhibitory activity assay

α-Glucosidase inhibitory activity assay was performed as reported previously [10] with minor modifications. α-Glucosidase was obtained from *Saccharomyces cerevisiae* and dissolved in potassium phosphate buffer (pH 6.8) to achieve a concentration of 0.26 U/mL. A total of 50 μL of the test sample (1–200 μg/mL extract, 0.01–2 mM pure compounds) was mixed with 50 μL of α-glucosidase. After incubation at 37°C for 10 min, 100 μL of the substrate, 4-nitrophenyl α-glucopyranoside (5 mM, in potassium phosphate buffer) was added to the mixture. The enzymatic reaction was allowed to proceed at 37°C for 20 min and stopped by addition of 100 μL of 0.2 M NaCO_3_. 4-Nitrophenol absorptions were subsequently measured at 405 nm using a spectrophotometer. The percentage inhibition of α-glucosidase was calculated as inhibition rate (%) = 100 × [1 - (*A*_sample_–*A*_s-blank_)/(*A*_control_–*A*_blank_)], where *A*_sample_ represents the absorbance of the reaction system containing the test sample, enzyme, and substrate, *A*_s-blank_ represents the absorbance of the reaction system containing the test sample and substrate but without enzyme, *A*_control_ represents the absorbance of the reaction system containing the enzyme and substrate but without test sample, and *A*_blank_ represents the absorbance of the reaction system containing the substrate but without test sample and enzyme. *A*_carbose_ was used as a positive control. The IC_50_ value was defined as the concentration of α-glucosidase inhibitor required to inhibit 50% of α-glucosidase activity under the assay conditions. Experiments were performed in triplicate, and all results are expressed as mean ± SEM.

## Results and Discussion

### Screening of α-glucosidase inhibitors from *P*. *tuberosa*

A method combining Sepbox chromatography and TLC bioautography was developed to identify α-glucosidase inhibitors from *P*. *tuberosa*. Sepbox chromatography separated a total of 150 subfractions (designated Pt1–Pt150) from the ethyl acetate fraction of *P*. *tuberosa* extracts within 20 h, after which modified TLC autographic assay was used to examine the α-glucosidase inhibitory activity of each subfraction. As shown in S1 Fig., compared with the previously reported TLC autographic assay [9], the modified assay showed a darker background color, thereby allowing easier detection of active spots. In the bioautographic chromatograms, both the ethyl acetate fraction of *P*. *tuberosa* extract and acarbose solution produced white spots, which indicates their α-glucosidase inhibitory activities. At least nine spots (corresponding to subfractions Pt15, Pt35, Pt76, Pt102, Pt103, Pt104, Pt120, Pt121, and Pt133) in the chromatograms showed α-glucosidase inhibitory activities.

Analyses of the HPLC data revealed that subfractions Pt15, Pt35, Pt102, and Pt120 contained only one major compound and the five other subfractions (Pt76, Pt103, Pt104, Pt121 and Pt133) contained less than four compounds. Pt15, Pt35, Pt102, and Pt120 were individually collected and concentrated, yielding 4.52 mg of compound **5**, 25.7 mg of compound **7**, 9.10 mg of compound **15**, and 4.83 mg of compound **4**, respectively. The five other subfractions were individually collected and further purified by preparative HPLC to produce compound **1** (11.24 mg), compound **2** (3.40 mg), compound **3** (3.01 mg), compound **6** (1.78 mg), compound **8** (4.96 mg), compound **9** (3.78 mg), compound **10** (2.11 mg), compound **11** (5.08 mg), compound **12** (6.01 mg), compound **13** (5.12 mg), compound **14** (9.15 mg), compound **16** (2.16 mg), compound **17** (5.62 mg), compound **18** (1.69 mg), compound **19** (1.32 mg), and compound **20** (3.14 mg). The origins of these compounds are shown in Table 1.

**Table 1:** Origins and α-glucosidase inhibitory activities of the pure isolated compounds.

Active fraction	Compound	IC_50_ (mM)
Pt15	5	0.482 ± 0.019
Pt35	7	0.518 ± 0.017
Pt76	8	1.203 ± 0.032
	9	-
	12	0.562 ± 0.021
Pt102	15	-
Pt103	1	0.379 ± 0.016
	2	0.624 ± 0.026
	3	0.721 ± 0.037
Pt104	6	0.210 ± 0.010
	14	-
	16	-
Pt120	4	0.067 ± 0.003
Pt121	17	0.229 ± 0.017
	18	0.283 ± 0.013
	19	0.255 ± 0.013
	20	0.371 ± 0.015
Pt133	10	-
	11	0.428 ± 0.018
	13	-
Positive control	Acarbose	3.72 ± 0.113

**Fig 1:**
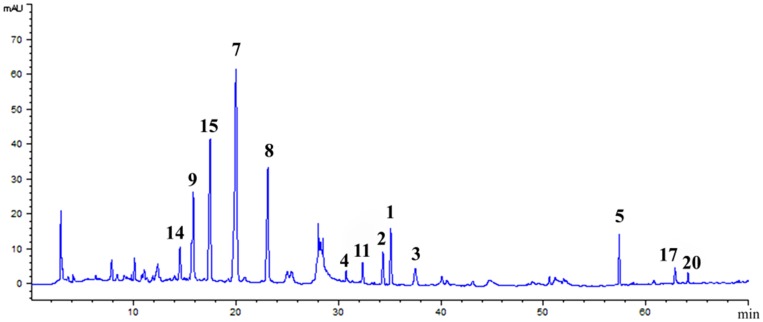
HPLC chromatogram of the ethyl acetate fraction of *P*. *tuberosa* extracts. Peaks with numbers represent isolated compounds.

HPLC analysis of the ethyl acetate fraction indicated that most of the major compounds shown in the HPLC profile could be separated by Sepbox chromatography (Fig. 1). Following HPLC, the purities of compounds **1**, **2**, **3**, **4**, **5**, **6**, **7**, **8**, **9**, **10**, **11**, **12**, **13**, **14**, **15**, **16**, **17**, **18**, **19**, and **20** were 99.1%, 98.3%, 97.7%, 92.8%, 96.6%, 92.4%, 98.1%, 92.5%, 91.8%, 95.3%, 94.1%, 92.5%, 92.7%, 98.2%, 97.3%, 92.5%, 98.5%, 91.1%, 92.0%, and 99.0%, respectively. This work is the first to report the simultaneous separation of 20 compounds from *P*. *tuberosa*.

### Structural determination of isolated compounds

The 20 isolated compounds were identified by ^1^H and ^13^C NMR analyses and compared with published data.

Compound **1** was obtained as a white amorphous powder; [α]^20^_D_ + 0.255 (*c* 1.8, MeOH); UV (MeOH) λ_max_ (log ε) 218 (1.78), 242 (1.11), 302 (0.46), and 362 (0.28) nm (S2 Fig.). Its molecular formula C_20_H_22_O_6_ indicated 11 degrees of unsaturation, as deduced from its HRESIMS spectrum at *m/z* 357.1329 ([M–H]^-^, calcd. 357.1338; S3 Fig.). The IR spectrum of **1** (S4 Fig.) showed hydroxyl (3399 cm^-1^), carbonyl (1745 and 1697 ^cm-1^), and phenyl (1587 and 1243 ^cm-1^) groups. The ^1^H NMR spectrum of **1** (S5 Fig.) showed two methyl singlets at δ_H_ 1.32 (3H, s, H-19) and δ_H_ 1.35 (3H, s, H-20) and one aldehyde proton at δ_H_ 10.9 (1H, s, H-15). The ^13^C NMR and DEPT spectra of **1** (S6 Fig.) indicated a total of 20 carbon signals comprising two methyls, six methylenes, one methine, one aldehyde, and ten quaternary carbons. The relevant ^1^H and ^13^C NMR data are given in Table 2. The spectroscopic characteristics described above resemble those of an abietane diterpene with a tetracyclic system. HMBC correlations between H-20 (δ_H_ 1.35) and C-1 (δ_C_ 40.7), C-5 (δ_C_ 54.8), C-9 (δ_C_ 151.5), and C-10(δ_C_ 42.1); between H-3 (δ_H_ 2.21) and C-1 (δ_C_ 40.7); and between H-19 (δ_H_ 1.32) and C-3 (δ_C_ 38.2), C-4 (δ_C_ 44.8), and C-5(δ_C_ 54.8) support the abietane diterpene skeleton. Correlations between the aldehyde group (δ_H_ 10.9) and C-9 (δ_C_ 151.5), C-11 (δ_C_ 131.8) were also observed in the HMBC spectrum (S8 Fig.). Thus, the structure of **1** was established as 14-hydroxyabieta-8,11,13-triene-11-carbaldehyde-18-oic-12-carboxy-13-(1-hydroxy-1-methylethyl)-lactone.

**Table 2:** ^1^H and ^13^C NMR data of compounds **1–4** in CD_3_OD (400 MHz and 100 MHz for ^1^H and ^13^C NMR, respectively; δ in ppm, ***J*** values in Hz).

Position	1		2		3		4	
	δ_H_	δ_C_	δ_H_	δ_C_	δ_H_	δ_C_	δ_H_	δ_C_
1	2.21 m, 1.28 td (13.2, 3.6)	40.7	2.38 d (12.6), 1.41 td (13.2, 3.0)	41.1	2.73 d (12.0), 1.43 m	37.5	1.91 m, 1.88 m	36.8
2	2.02 m, 1.55 m	21.1	2.06 m, 2.11 m	21.2	2.08 m, 1.63 d (14.0)	20.9	1.26 m	38.2
3	2.21 d (13.2), 1.08 td (13.8, 4.2)	38.2	2.26 d (13.2), 1.13 td (13.2, 3.6)	38.6	2.22 d (12.8), 1.12 td (13.6, 3.6)	38.4	1.73 m, 1.66 m	19.7
4	-	44.8	-	44.8	-	45.0	-	38.4
5	1.44 d (11.4)	54.8	1.55 d (12.6)	53.2	1.52 d (12.4)	53.6	1.31 dd (12.8, 1.6)	54.1
6	2.32 dd (14.4, 7.2), 2.03 m	20.6	2.33 dd (13.8, 6.6), 1.65 d (13.8)	21.3	2.32 dd (14.0, 7.2), 2.13 m	20.8	1.65 m, 1.47 m	20.3
7	3.04 dd (18.6, 4.8), 2.63 ddd (19.8, 13.2, 7.2)	28.6	3.08 dd (18.0, 4.8), 2.57 ddd (18.6, 12.6, 6.6)	27.5	3.06 dd (18.8, 5.6), 2.58 ddd (19.3, 12.6, 7.3)	28.2	2.04 dd (11.2, 6.8), 1.98 d (6.4)	34.9
8	-	132.8	-	131.6	-	134.0	-	127.5
9	-	151.5	-	153.3	-	147.2	-	141.2
10	-	42.1	-	40.3	-	41.6	-	40.0
11		131.8	7.41 s	114.7	6.73 s	99.9	2.26 m, 2.11 m	30.2
12	-	130.1	-	129.9	-	121.2	2.44 m	26.8
13	-	124.8	-	125.0	-	123.2		126.8
14	-	152.0	-	150.2	-	157.9	6.31 d (0.8)	111.7
15	10.9 s	198.8	-	-	-	-	7.37 t (1.6)	143.9
16	-	172.4	-	174.3	-	171.5	7.27 s	139.6
17	5.30 d (15.0), 5.27 d (15.0)	69.6	5.26 s	69.6	5.35 d (14.4), 5.29 d (14.4)	59.0	1.60 s	19.8
18	-	181.1	-	181.3	-	181.5	4.24 d (9.6), 3.80 d (9.2)	71.8
19	1.32 s	29.5	1.34 s	29.2	1.32 s	29.6	1.02 s	27.7
20	1.35 s	21.4	1.20 s	23.9	1.35 s	21.3	0.99 s	21.2

Compound **2** was obtained as a white amorphous powder; [α]^20^_D_ + 0.087 (*c* 1.0, MeOH); UV (MeOH) λ_max_ (log ε) 210 (1.73), 254 (0.62), and 296 (0.29) nm (S9 Fig.). Its molecular formula C_19_H_22_O_5_ was determined based on its HRESIMS spectrum at *m*/*z* 329.1370 ([M–H]^-^, calcd. 329.1362; S10 Fig.). The IR spectrum of **2** (S11 Fig.) showed hydroxyl (3429 cm^1^), carbonyl (1735 and 1686 ^cm-1^), and phenyl (1591 and 1262 ^cm-1^) groups. ^1^H and ^13^C NMR data (Table 2; S12 and S13 Figs.) revealed the presence of two methyls [δ_H_ 1.20 (3H, s, H-20) and δ_H_ 1.34 (3H, s, H-19)], one lactone group [δ_C_ 174.3 (C-16)], one carboxylic group [δ_C_ 181.3 (C-18)], and one aromatic proton [δ_H_ 7.41 (1H, s) and δ_C_ 114.7]. These data, together with other spectroscopic characteristics, suggested that **2** is an abietane diterpenoid. HMBC correlations between H-20 (δ_H_ 1.20) and C-1 (δ_C_ 41.1), C-9 (δ_C_ 153.3), and C-10 (δ_C_ 40.3) supported this inference; the only difference between **1** and **2** is that the proton at C-11 in **2** is replaced by an aldehyde group in **1**, as deduced from HMBC correlations of H-11 (δ_H_ 7.41) with C-8 (δ_C_ 131.6), C-10 (δ_C_ 40.3), C-12 (δ_C_ 129.9), C-14 (δ_C_ 150.2), and C-16 (δ_C_ 174.3) (S15 Fig.). Thus, **2** was determined to be an abietane norditerpene, namely, 14-hydroxyabieta-8,11,13-triene-17-oic-12-carboxy-13-(1-hydroxy-1-methylethyl)-lactone.

Compound **3** was obtained as a yellow amorphous powder; [α]^20^_D_ + 0.3 (*c* 2.3, MeOH); UV (MeOH) λ_max_ (log ε) 217 (1.12), 257 (0.29), and 306 (0.20) nm (S16 Fig.). Its molecular formula was determined from its HRESIMS spectrum at *m*/*z* 349.2349 to be C_19_H_24_O_6_ ([M + H]^+^, calcd. 349.2355; S17 Fig.), which is 18 mass units larger than that of **2**; the difference in molecular formulas observed corresponds to one additional oxygen atom and two hydrogen atoms. The IR spectrum of **3** (S18 Fig.) showed hydroxyl (3434 ^cm-1^), carbonyl (1735 and 1654 ^cm-1^), and phenyl (1466 and 1287 ^cm-1^) groups. ^1^H and ^13^C NMR data (Table 2; S19 and S20 Figs.) revealed the presence of two methyls [δ_H_ 1.35 (3H, s, H-20), and δ_H_ 1.32 (3H, s, H-19)], one lactone group [δ_C_ 171.5 (C-16)], one carboxylic group [δ_C_ 181.5 (C-18)], one aromatic hydrogen δ_H_ 6.73 (1H, s), and carbon signals at δ_C_ 99.9. HMBC correlations between H-20 (δ_H_ 1.35) and C-1 (δ_C_ 37.5), C-9 (δ_C_ 147.2), and C-10 (δ_C_ 41.6) and between H-11 (δ_H_ 6.73) and C-8 (δ_C_ 134.0), C-10 (δ_C_ 41.6), C-12 (δ_C_ 121.2), C-14 (δ_C_ 157.9), and C-16 (δ_C_ 171.5) confirmed the tetracyclic abietane diterpene skeleton of **3**. HMBC correlations from H-17 (δ_H_ 5.35, 5.29) to C-12 (δ_C_ 121.2), C-13 (δ_C_ 123.2), C-14 (δ_C_ 157.9), and C-16 (δ_C_ 171.5) were also observed (S23 Fig.). According to the ^1^H and ^13^C NMR data shown in Table 2, **3** is very similar to **2**. Therefore, **3** was identified as 14,16-dihydroxyabieta-8,11,13-triene-15,17-dioic acid.

Compound **4** was obtained as a white amorphous powder; [α]^20^_D_ + 0.005 (*c* 0.68, MeOH); UV (MeOH) λ_max_ (log ε) 204 (0.22) nm (S24 Fig.). Its molecular formula was determined to be C_20_H_30_O_2_ by its HRESIMS spectrum at *m*/*z* 303.3067 ([M + H]^+^, calcd. 303.3059; S25 Fig.). The IR spectrum of **4** (S26 Fig.) showed hydroxyl (3432 ^cm-1^) and carbonyl (1655 and 1630 ^cm-1^) groups. The ^1^H NMR spectrum of **4** (S27 Fig.) showed three methyl singlets at δ_H_ 0.99 (3H, s, H-20), 1.02 (3H, s, H-19), and 1.60 (3H, s, H-17). ^13^C NMR and DEPT spectra (S28 Fig.) indicated a total 20 carbon signals composing three methyls, seven methylenes, three methines, one oxygen-bearing methylene, and five quaternary carbons. The relevant ^1^H and ^13^C NMR data are given in Table 2. The spectroscopic characteristics described above resemble those of a labdane diterpene with a tricyclic system [11]. HMBC correlations between H-20 (δ_H_ 0.99) and C-5 (δ_C_ 54.1), C-9 (δ_C_ 141.2), and C-10(δ_C_ 40.0); between H-19 (δ_H_ 1.02) and C-2 (δ_C_ 38.2), C-4 (δ_C_ 38.4), and C-5(δ_C_ 54.1); between H-17 (δ_H_ 1.60) and C-7 (δ_C_ 34.9), C-8(δ_C_ 127.5), and C-9 (δ_C_ 141.2); between H-11 (δ_H_ 2.26, 1.29) and C-8 (δ_C_ 127.5), C-9 (δ_C_ 141.2), and C-10(δ_C_ 40.0); between H-3 (δ_H_ 1.73, 1.66) and C-19 (δ_C_ 27.7); and between H-18 (δ_H_ 4.24, 3.80) and C-4 (δ_C_ 38.5), C-19 (δ_C_ 27.7), and C-5(δ_C_ 54.1) supported the labdane diterpene skeleton. Correlations between H-14 (δ_H_ 6.31) and C-13 (δ_C_ 126.8); between H-15 (δ_H_ 7.37) and C-13 (δ_C_ 126.8); and between H-16 (δ_H_ 7.27) and C-13 (δ_C_ 126.8) were also observed in the HMBC spectrum (S30 Fig.). Thus, **4** was determined to be 15,16-eposy-8,13(16),14-labdatrien-19-ol and named phlomisol.

**Fig 2:**
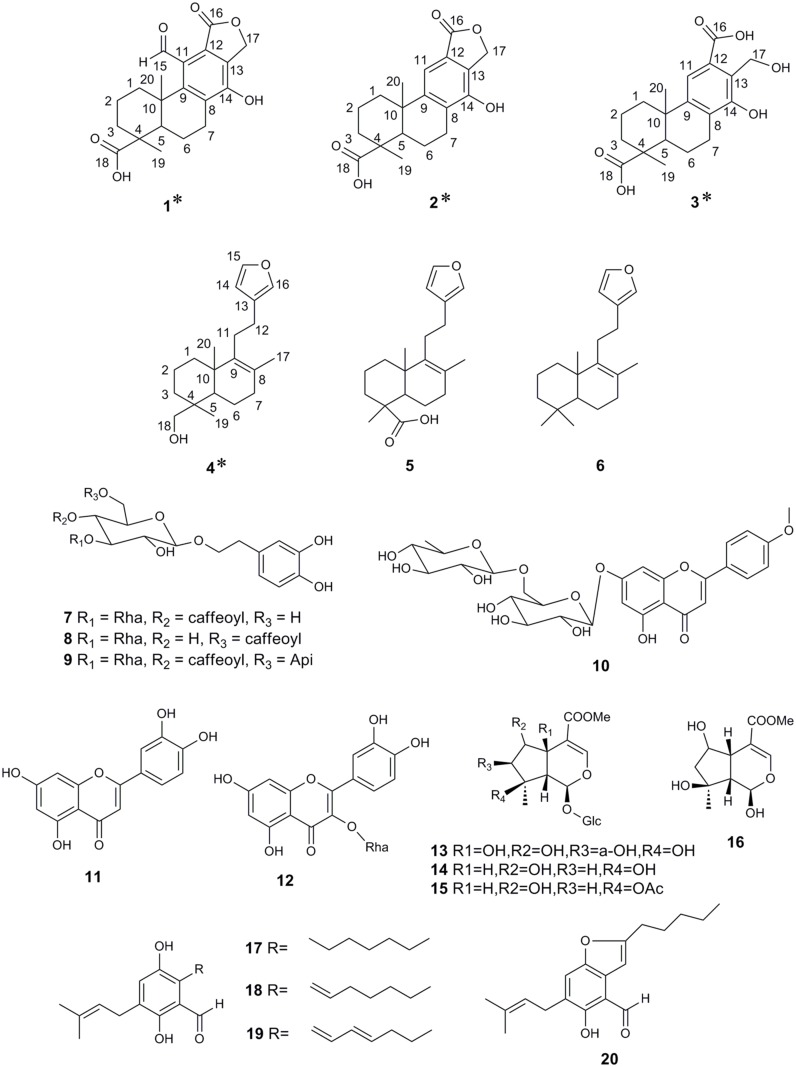
Structures of compounds 1–20 isolated from *P*. *tuberosa*. Asterisks indicate new compounds.

Comparison of physical and spectral findings with published data allowed identification of compounds **5** to **20** as phlomisoic acid (**5**) [11], 15,16-eposy-8,13(16),14-labdatrien (**6**) [12], acteoside (**7**), isoacteoside (**8**) [13], forsythoside B (**9**) [14], acacetin-7-rutinoside (**10**) [15], luteolin (**11**) [16], quercetin-3-rhamnoside (**12**) [17], phlomiol (**13**) [18], shanzhiside methyl ester (**14**), 8-O-acetylshanzhiside methyl ester (**15**) [19], 8-O-acetylshanzhigenin methyl ester (**16**) [20], flavoglaucin (**17**), tetrahydroauroglaucin (**18**), dihydroauroglaucin (**19**) [21], 2-(2′,3-epoxy-1′-heptenyl)-6-hydroxy-5-(3″-methyl-2″-butenyl) benzaldehyde (**20**) [22]. The structures of the 20 isolated compounds are shown in Fig. 2. Compounds **1** to **4** are original natural compounds, compounds **6**, **10**, and **17**–**20** are isolated from the genus *Phlomis* for the first time, and compounds **5**, **8**, **11**–**13**, **15**, and **16** are isolated from *P*. *tuberosa* for the first time.

### α-Glucosidase inhibitory activity of the isolated compounds

Another TLC bioautographic assay was conducted to confirm the α-glucosidase inhibitory activity of the isolated compounds. The ethyl acetate fraction of *P*. *tuberosa* extracts and 20 individual pure compounds were migrated with the appropriate solvent, inspected under UV 366 nm, and then subjected to bioautographic assay (S31 Fig.). In the bioautographic chromatograms, 15 of the compounds, including compounds **1**–**8**, **10**–**12**, and **17**–**20**, were active components corresponding to the active spots of the ethyl acetate fraction of *P*. *tuberosa* extracts. This result indicates that the α-glucosidase inhibitory components of *P*. *tuberosa* can be obtained by an integrated Sepbox chromatography prefractionation and TLC bioautograph-guided separation strategy. However, we note that the data in S31 Fig. are not quantitative.

The inhibitory capacity of these compounds was quantitatively estimated by conventional α-glucosidase inhibitory activity assay because this method is considered to be the most direct and reliable method for determining α-glucosidase inhibitory capacity thus far [10]. Results showed that compounds **1**–**8**, **10**–**12**, and **17**–**20** exhibited much stronger α-glucosidase inhibitory activities (with IC_50_ values in the range 0.067–1.203 mM) than the positive control acarbose (IC_50_ = 3.72 ± 0.113 mM; Table 1). Previous toxicity studies have also demonstrated that compounds **7**, **10**, and **12** are nontoxic [23–25], which indicates their potential use as antidiabetes drugs. Toxicological tests of other compounds have yet to be performed.

## Conclusions

A simple, rapid, and effective Sepbox chromatography method coupled to TLC bioautography was established to probe active ingredients with α-glucosidase inhibition capability from *P*. *tuberosa*. Twenty compounds, including four new compounds, were isolated; six of these compounds were first isolated from the genus *Phlomis*. Among the compounds obtained from *P*. *tuberosa*, the four new compounds and 11 other compounds showed significant α-glucosidase inhibition activities. These compounds demonstrated much higher α-glucosidase inhibitory capacities than acarbose, which indicates their potential use as alternative medicines for diabetes mellitus.

## Supporting Information

S1 Fig Fraction detection and screening of the α-glucosidase inhibitory activity of *P*.*tuberosa*. Pt: ethyl acetate fraction of *P*. *tuberosa* (5 mg/mL, 20 μL); Acar: acarbose (1 mg/mL, 4 μL); 1–150: 150 subfractions (20 μL) obtained by Sepbox chromatography separation. Subfractions with α-glucosidase inhibitory activity are boxed in yellow frames.
(TIF)

S2 Fig UV spectrum (MeOH) of 1. (TIF)

S3 Fig HRESIMS spectrum of 1. (TIF)

S4 Fig IR (KBr, disc) spectrum of 1. (TIF)

S5 Fig ^1^H NMR spectrum (MeOD, 600 MH_Z_) of 1. (TIF)

S6 Fig ^13^C NMR and DEPT spectra (MeOD, 150 MH_Z_) of 1. (TIF)

S7 Fig HSQC spectrum of 1. (TIF)

S8 Fig HMBC spectrum of 1. (TIF)

S9 Fig UV spectrum (MeOH) of 2. (TIF)

S10 Fig HRESIMS spectrum of 2. (TIF)

S11 Fig IR (KBr, disc) spectrum of 2. (TIF)

S12 Fig ^1^H NMR spectrum (MeOD, 400 MH_Z_) of 2. (TIF)

S13 Fig ^13^C NMR and DEPT spectra (MeOD, 100 MH_Z_) of 2. (TIF)

S14 Fig HSQC spectrum of 2. (TIF)

S15 Fig HMBC spectrum of 2. (TIF)

S16 Fig UV spectrum (MeOH) of 3. (TIF)

S17 Fig HRESIMS spectrum of 3. (TIF)

S18 Fig IR (KBr, disc) spectrum of 3. (TIF)

S19 Fig ^1^H NMR spectrum (CDCl_3_, 400 MH_Z_) of 3. (TIF)

S20 Fig ^13^C NMR and DEPT spectra (CDCl_3_, 100 MH_Z_) of 3. (TIF)

S21 Fig COSY spectrum of 3. (TIF)

S22 Fig HSQC spectrum of 3. (TIF)

S23 Fig HMBC spectrum of 3. (TIF)

S24 Fig UV spectrum (MeOH) of 4. (TIF)

S25 Fig HRESIMS spectrum of 4. (TIF)

S26 Fig IR (KBr, disc) spectrum of 4. (TIF)

S27 Fig ^1^H NMR spectrum (CDCl_3_, 400 MH_Z_) of 4. (TIF)

S28 Fig ^13^C NMR and DEPT spectra (CDCl_3_, 100 MH_Z_) of 4. (TIF)

S29 Fig HSQC spectrum of 4. (TIF)

S30 Fig HMBC spectrum of 4. (TIF)

S31 Fig Detection of the α-glucosidase inhibitory activities of 20 isolated compounds. Twenty microliters of the ethyl acetate fraction (Pt, 5 mg/mL) of *P*. *tuberosa* and 4 μL of the pure isolates (2.5 mM) were applied as bands on TLC plates. The first plate was eluted with petroleum ether/ethyl acetate (10:1), the second plate was eluted with trichloromethane/methanol (12:1), and the third plate was eluted with ethyl acetate/methanol/water (15:2:1).
(TIF)

S1 Table Fractionation conditions of the ethyl acetate extract of *Phlomis tuberosa* using the Sepbox system. (DOC)

S2 Table Solvent gradient program for HPLC analysis. (DOC)
