# Supplementary figures and images for: Rapid Identification of α-Glucosidase Inhibitors from Phlomis tuberosa by Sepbox Chromatography and Thin-Layer Chromatography Bioautography
Source: PLoS One. 2015 Feb 6;10(2):e0116922. doi: 10.1371/journal.pone.0116922 (PMC4319760; doi:10.1371/journal.pone.0116922)

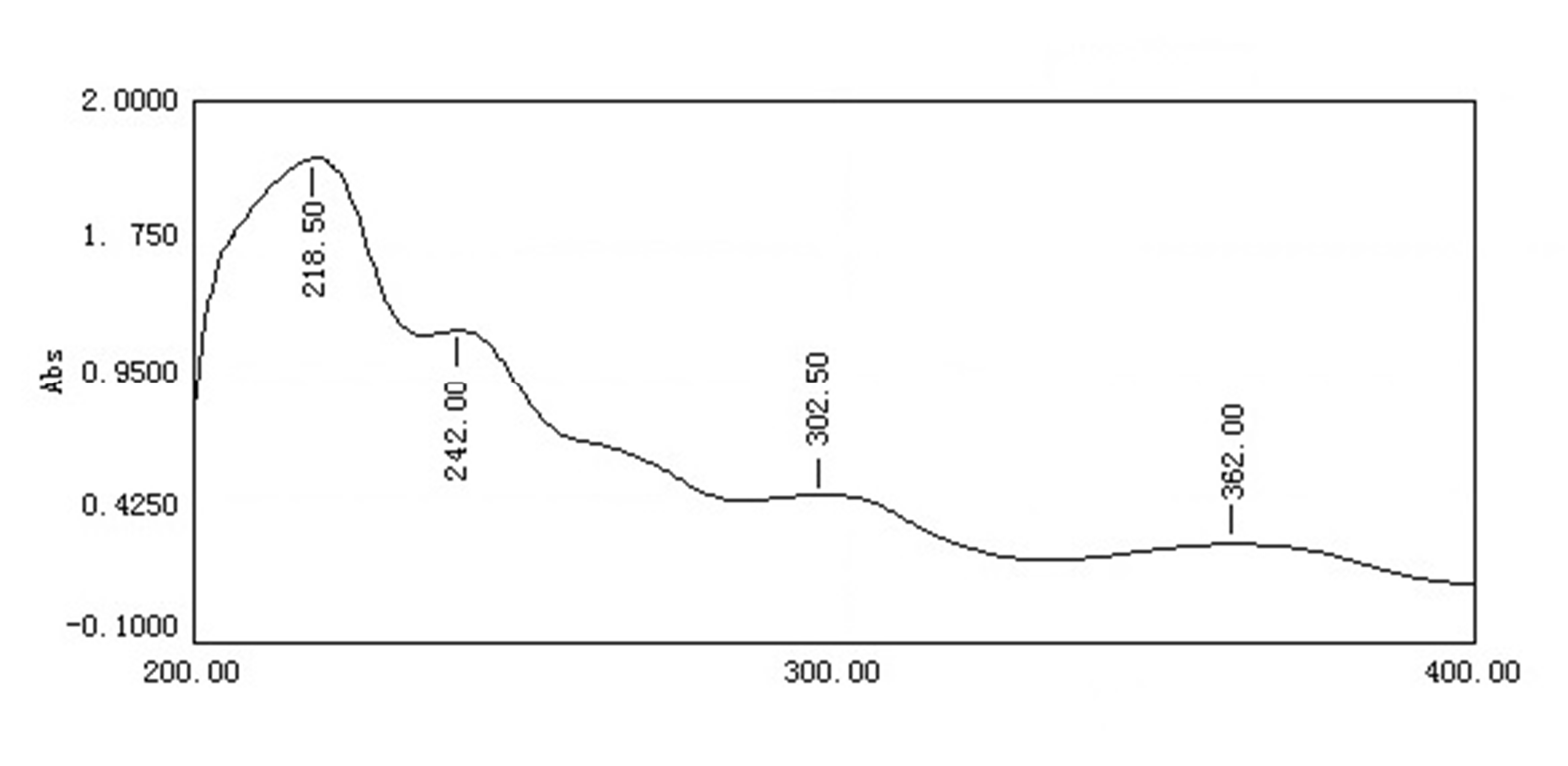

Supplement: S2 Fig — (TIF) [file pone.0116922.s002.tif]

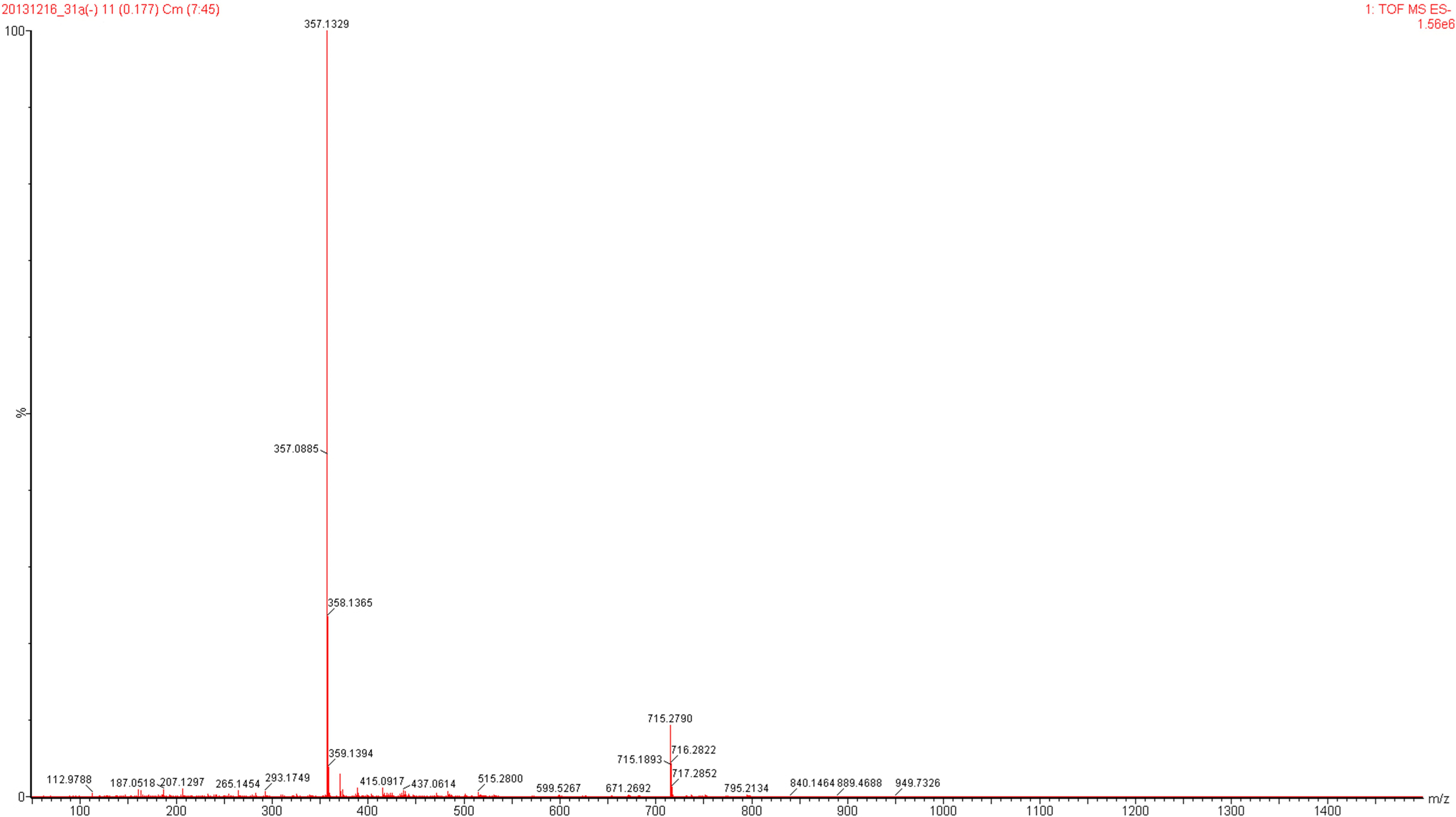

Supplement: S3 Fig — (TIF) [file pone.0116922.s003.tif]

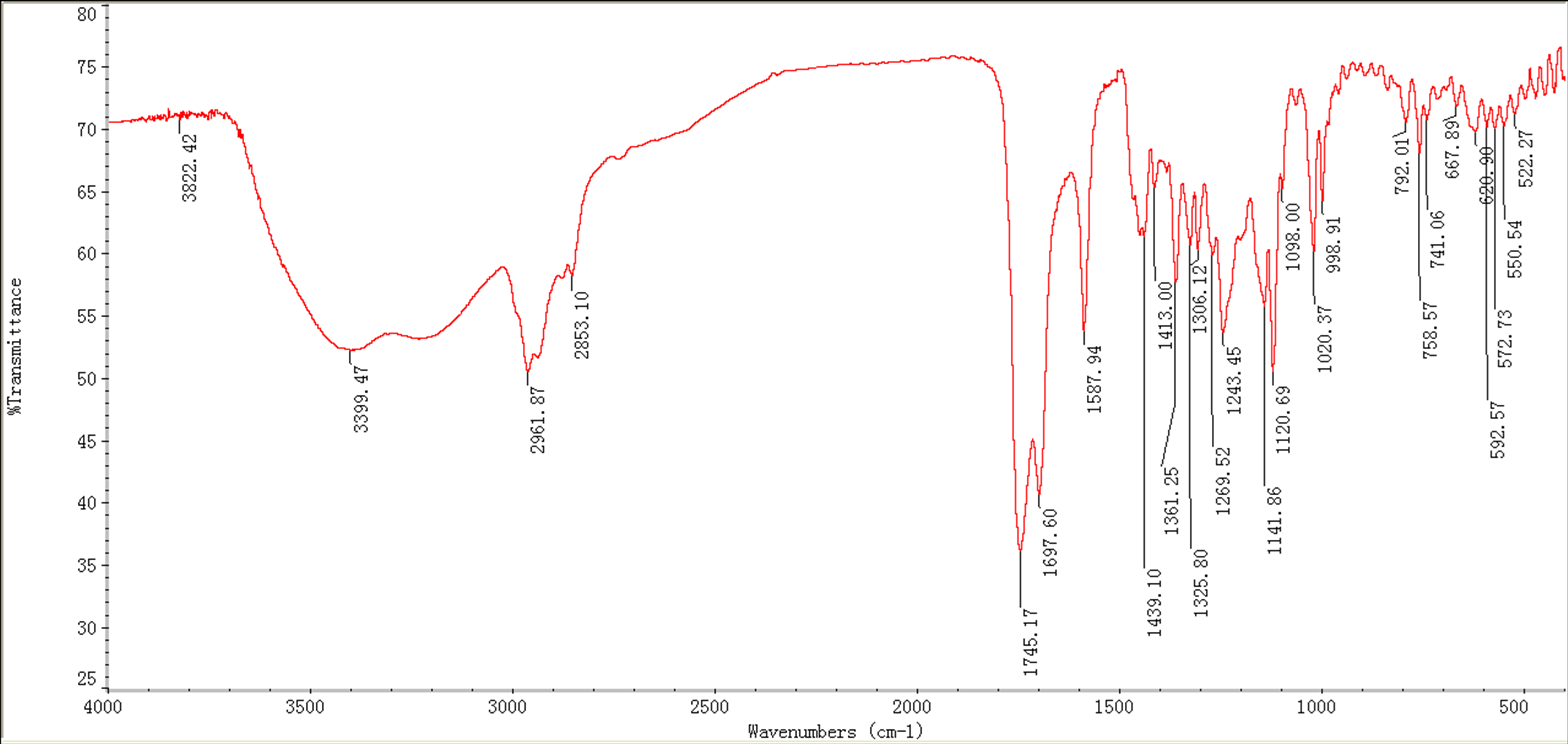

Supplement: S4 Fig — (TIF) [file pone.0116922.s004.tif]

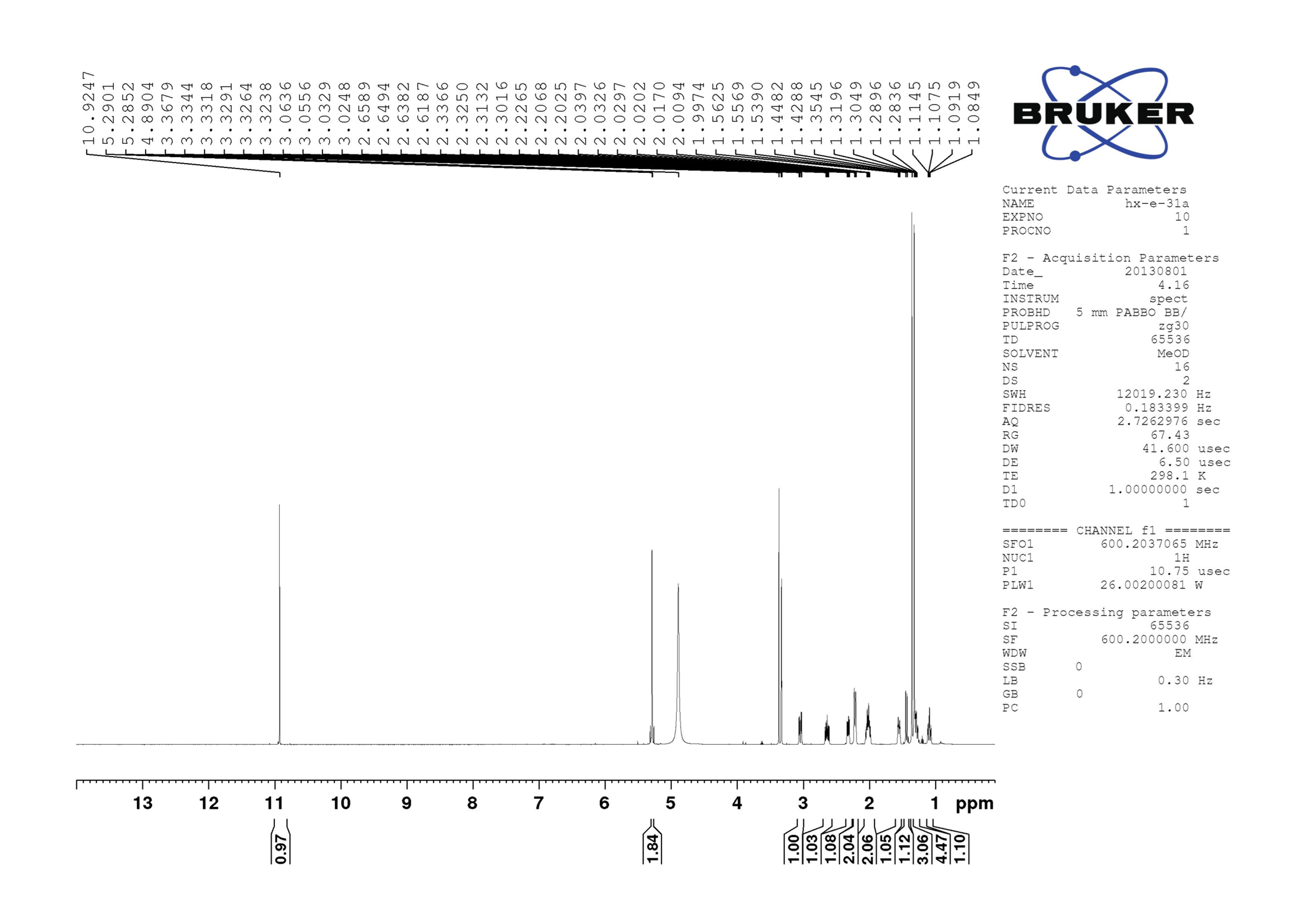

Supplement: S5 Fig — (TIF) [file pone.0116922.s005.tif]

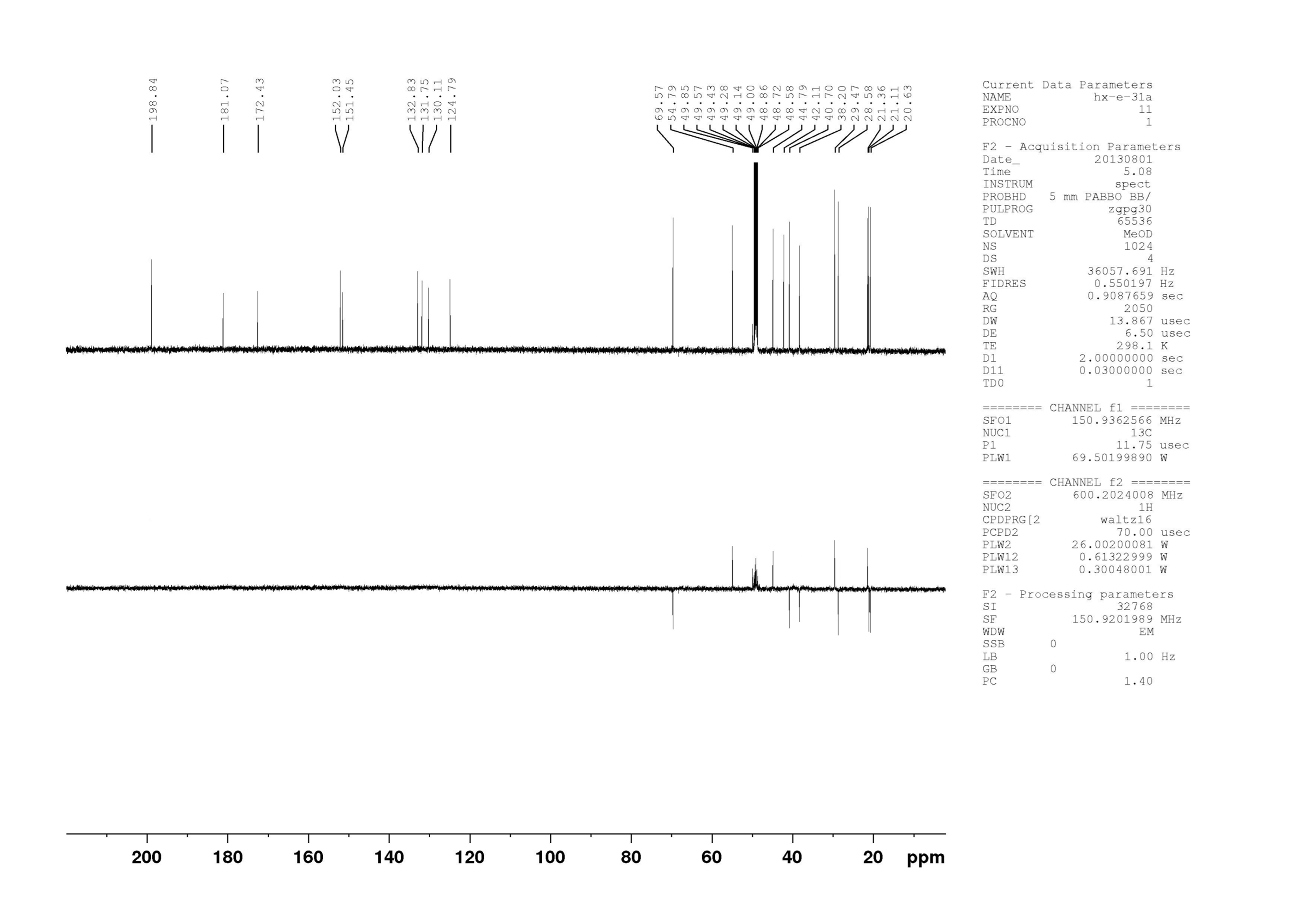

Supplement: S6 Fig — (TIF) [file pone.0116922.s006.tif]

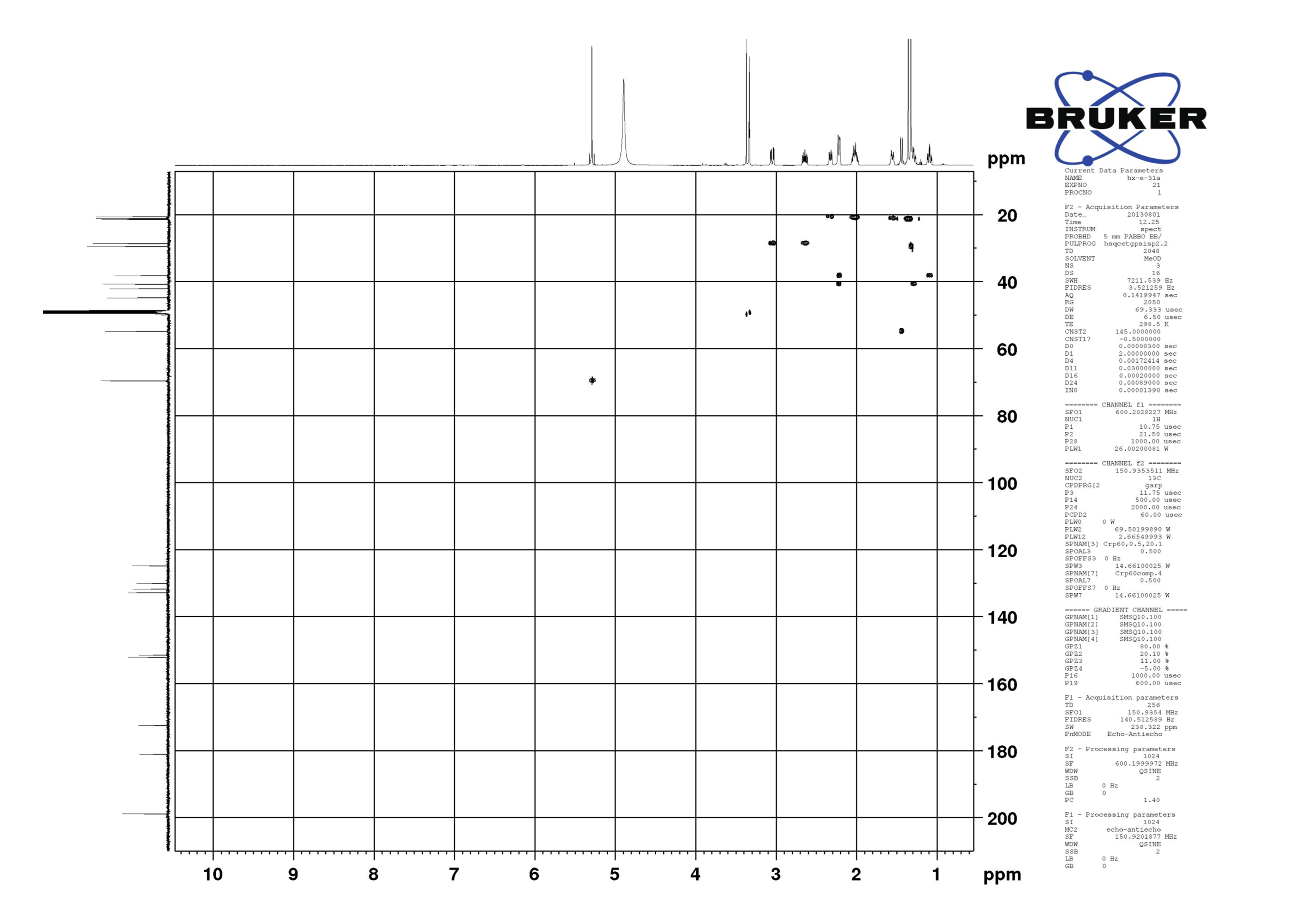

Supplement: S7 Fig — (TIF) [file pone.0116922.s007.tif]

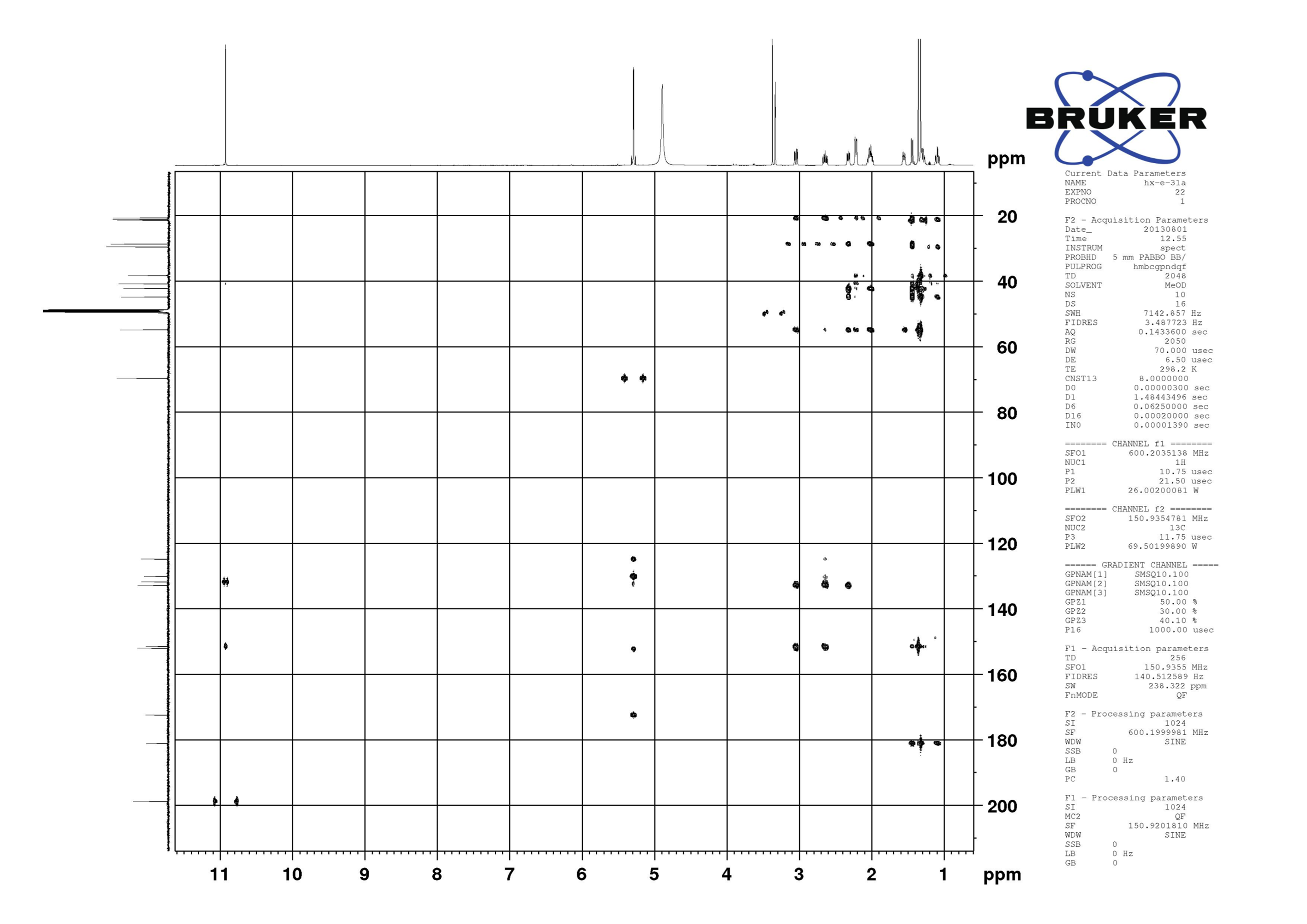

Supplement: S8 Fig — (TIF) [file pone.0116922.s008.tif]

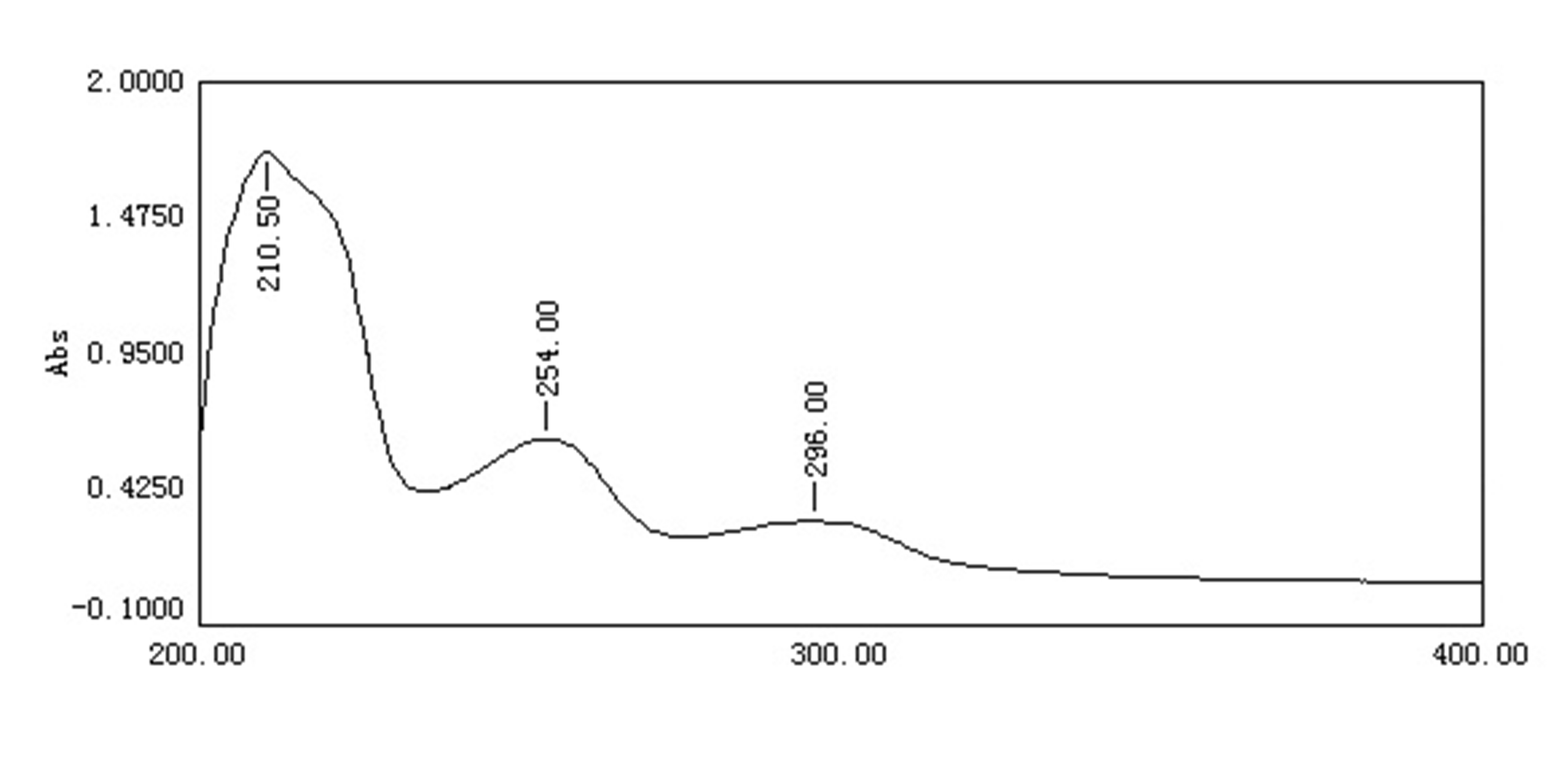

Supplement: S9 Fig — (TIF) [file pone.0116922.s009.tif]

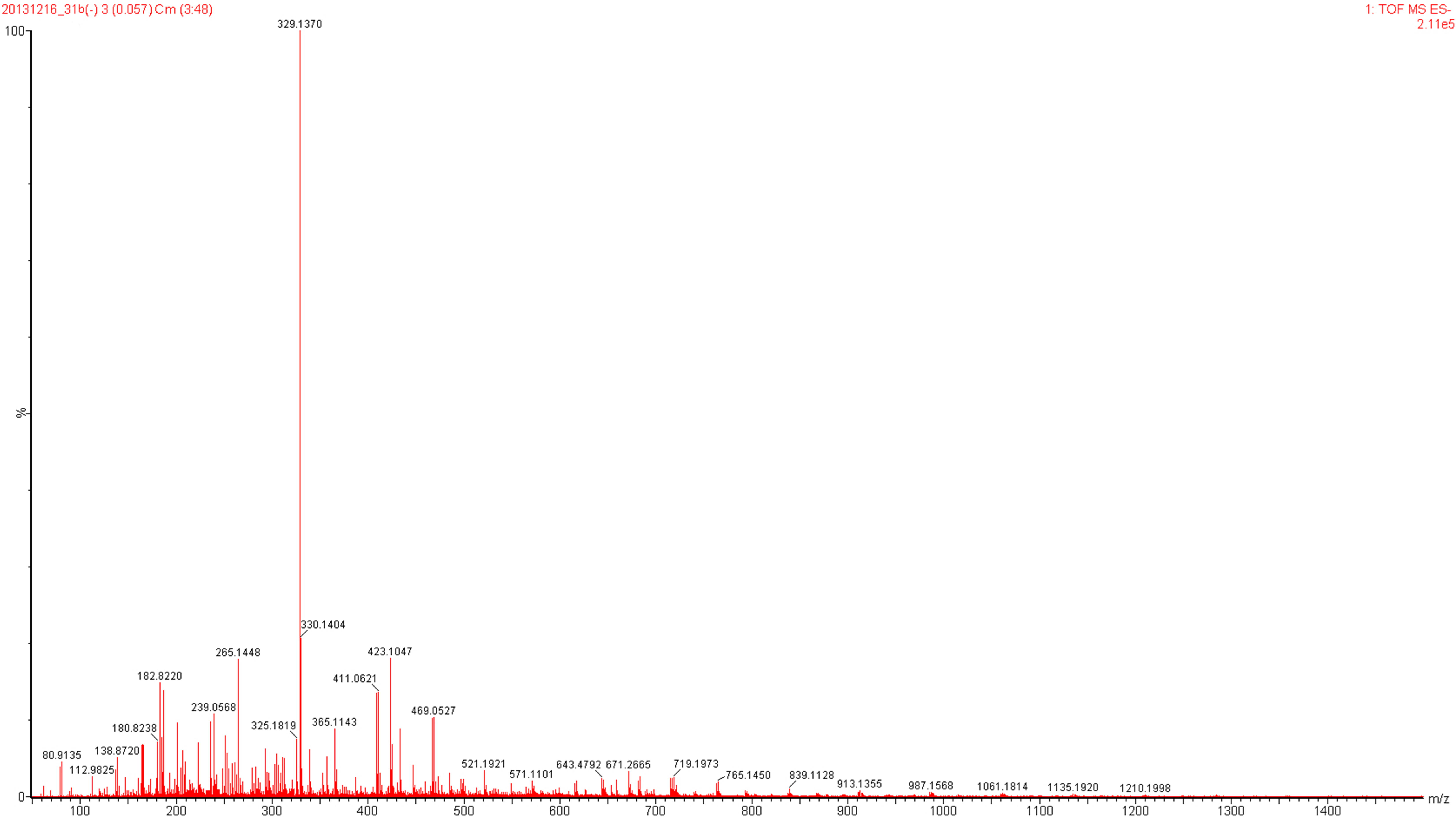

Supplement: S10 Fig — (TIF) [file pone.0116922.s010.tif]

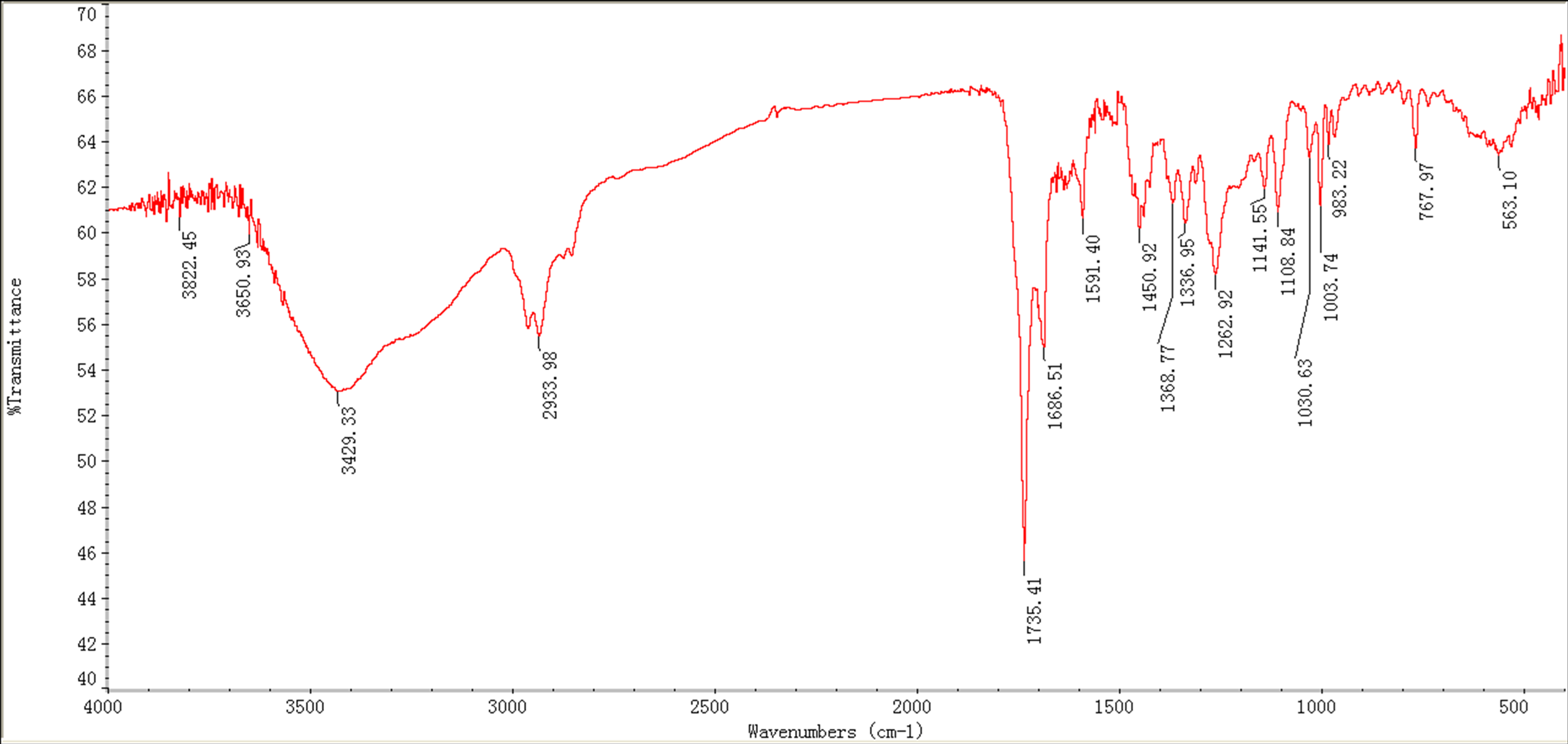

Supplement: S11 Fig — (TIF) [file pone.0116922.s011.tif]

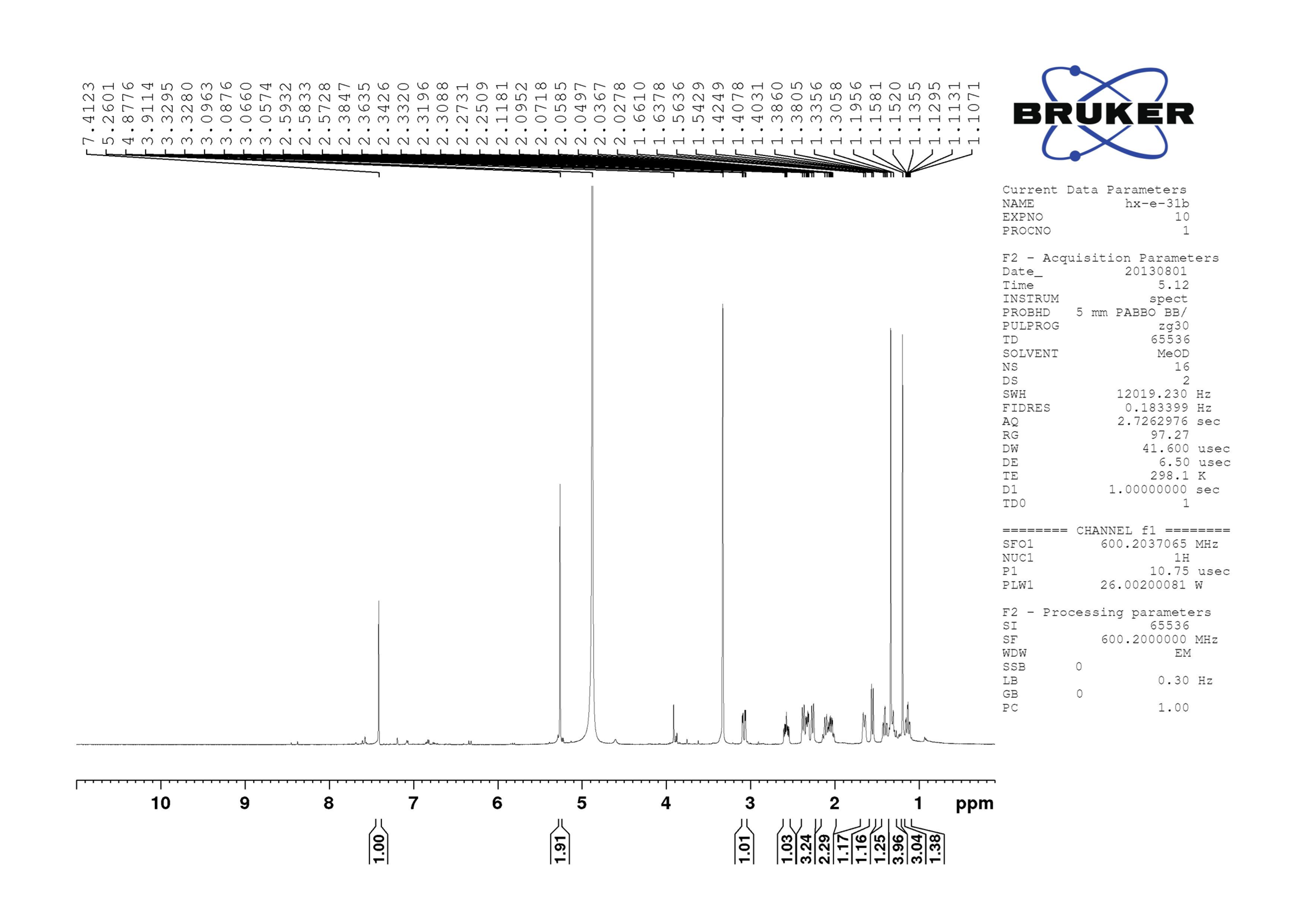

Supplement: S12 Fig — (TIF) [file pone.0116922.s012.tif]

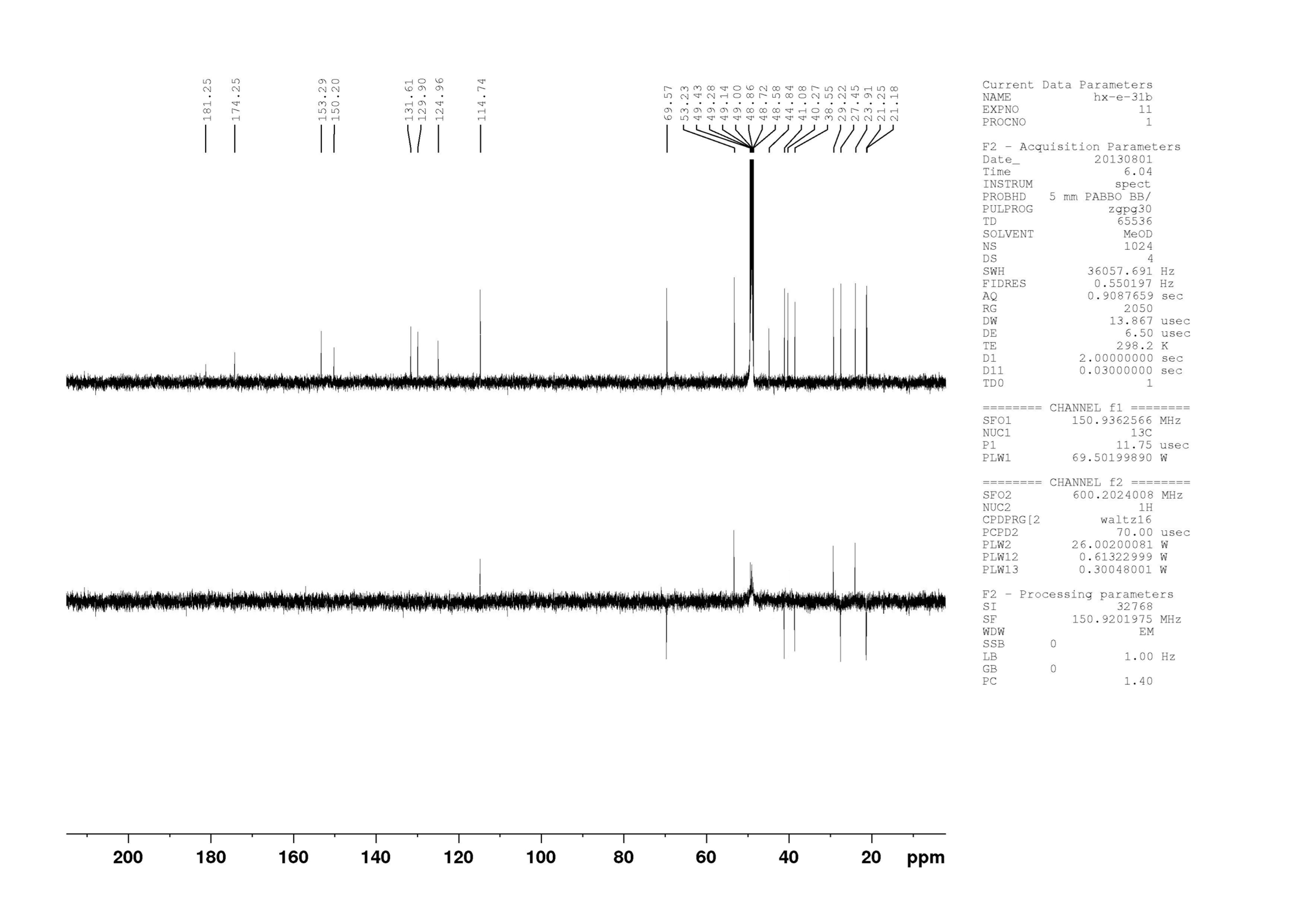

Supplement: S13 Fig — (TIF) [file pone.0116922.s013.tif]

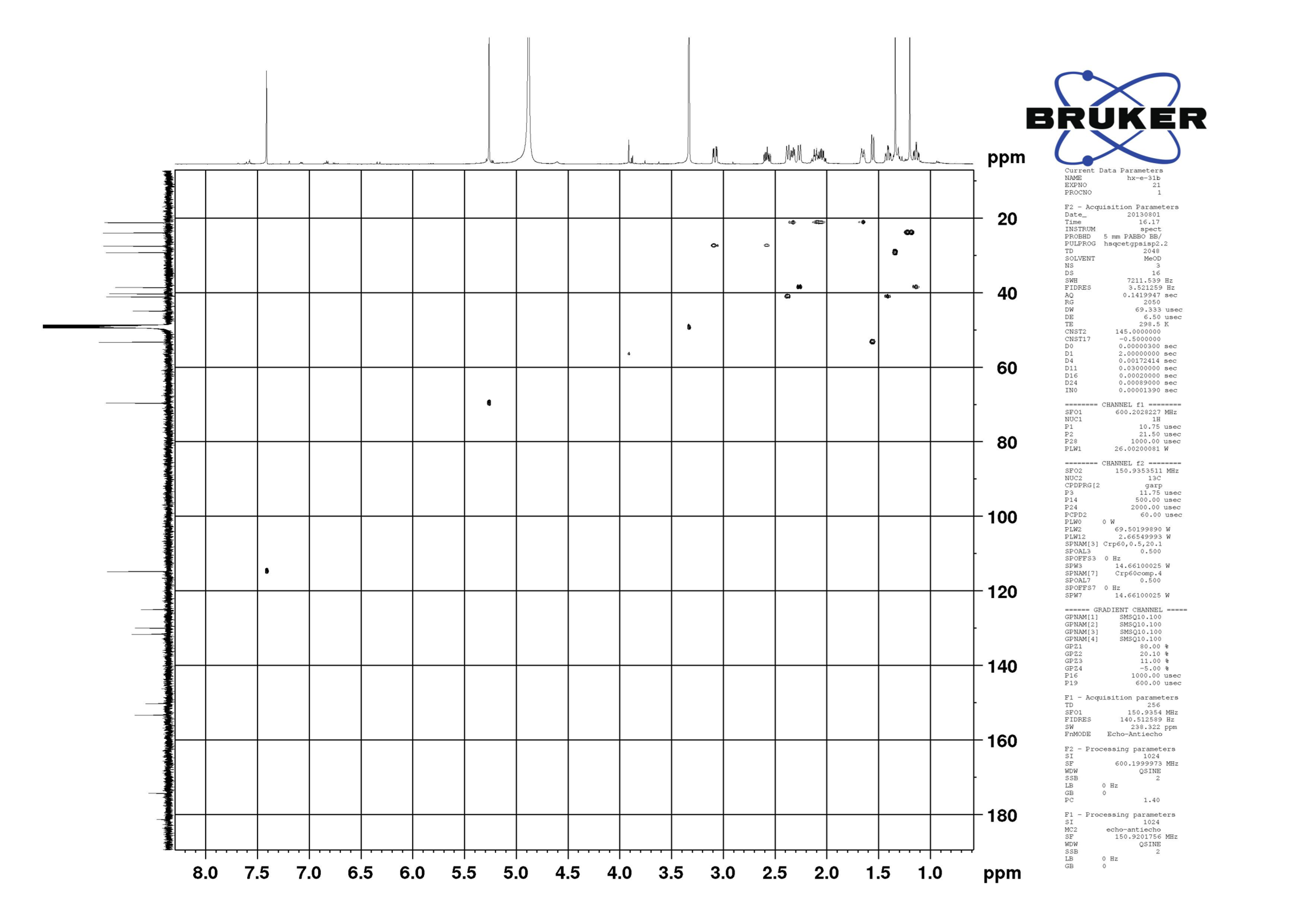

Supplement: S14 Fig — (TIF) [file pone.0116922.s014.tif]

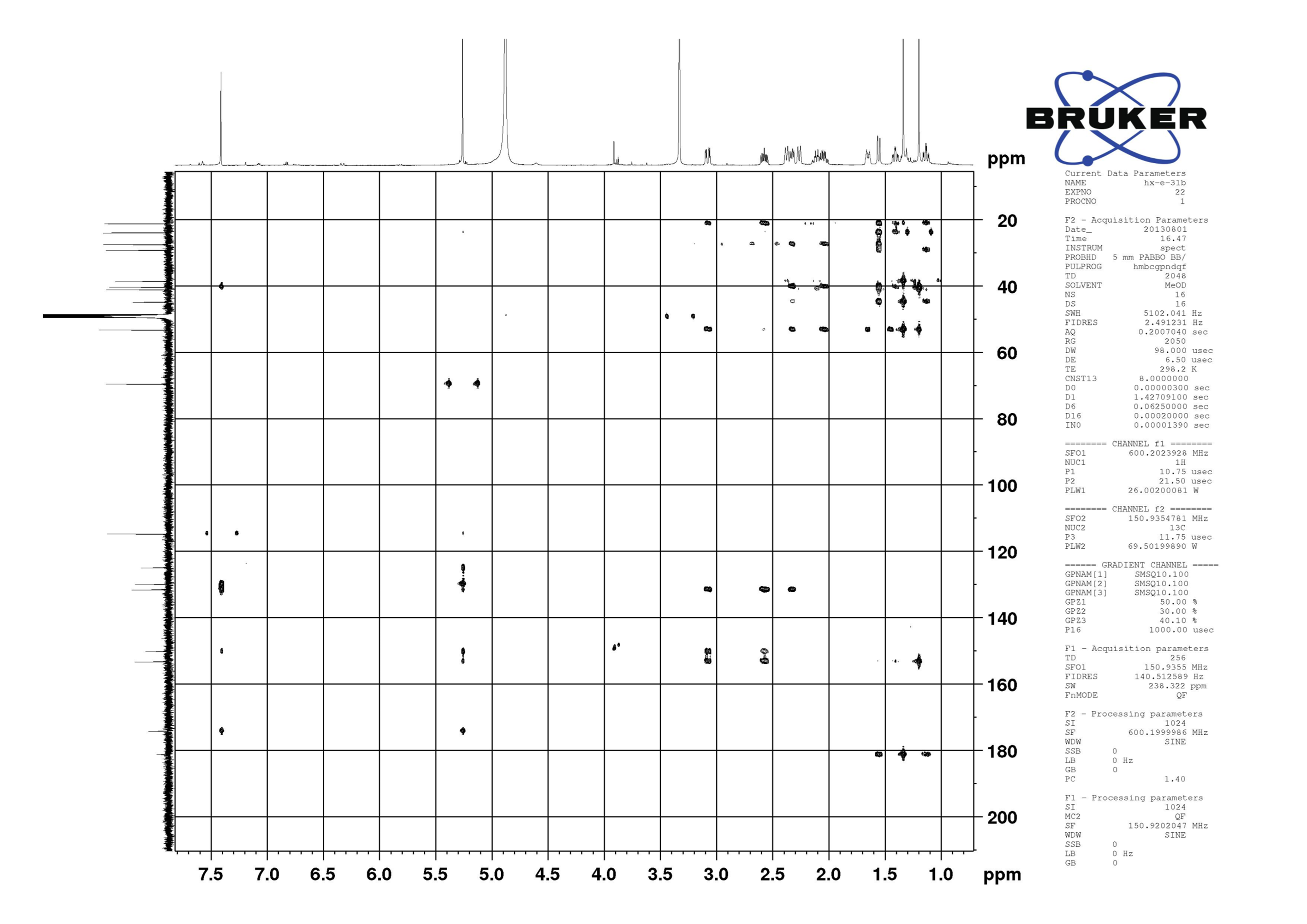

Supplement: S15 Fig — (TIF) [file pone.0116922.s015.tif]

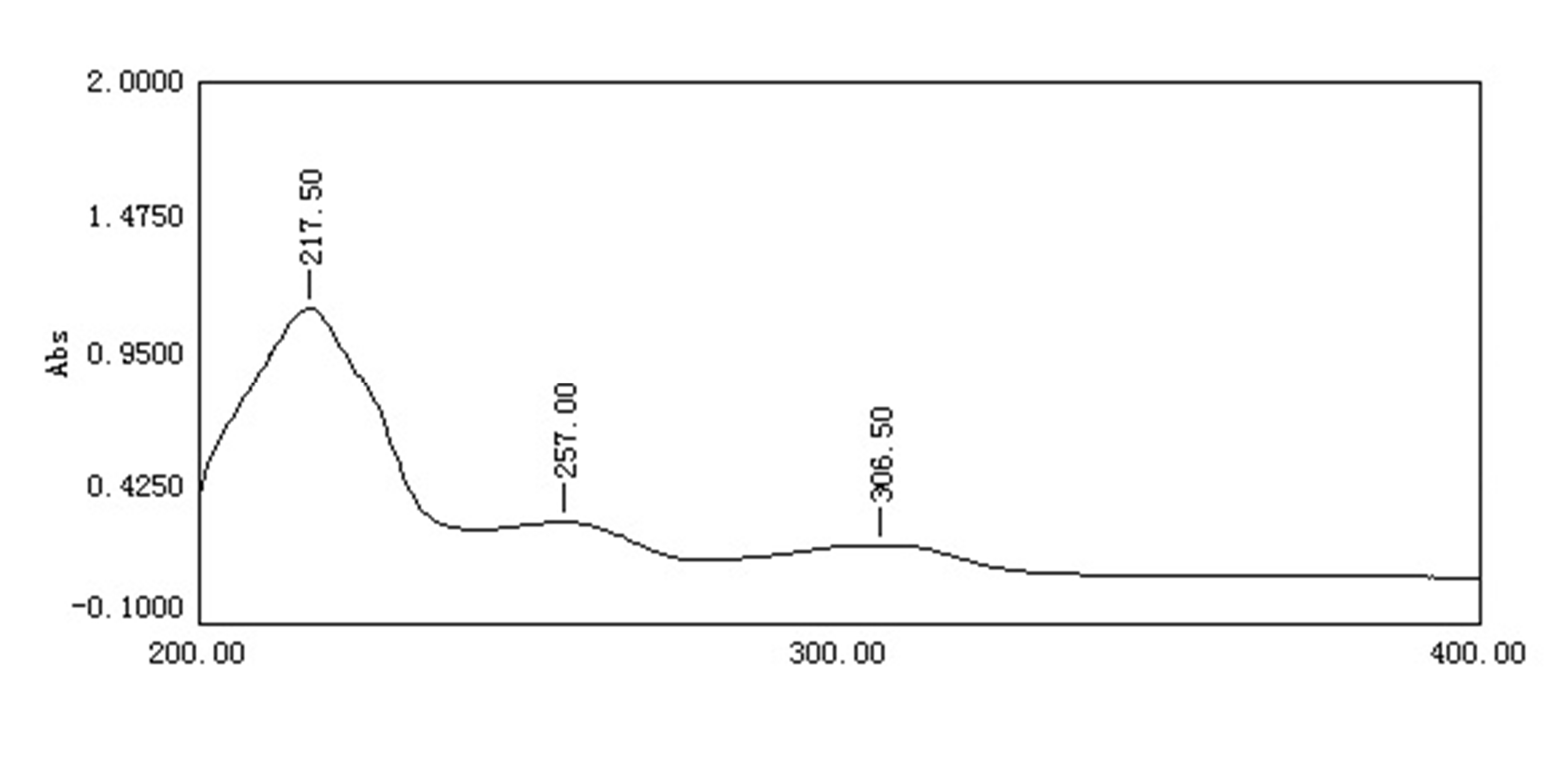

Supplement: S16 Fig — (TIF) [file pone.0116922.s016.tif]

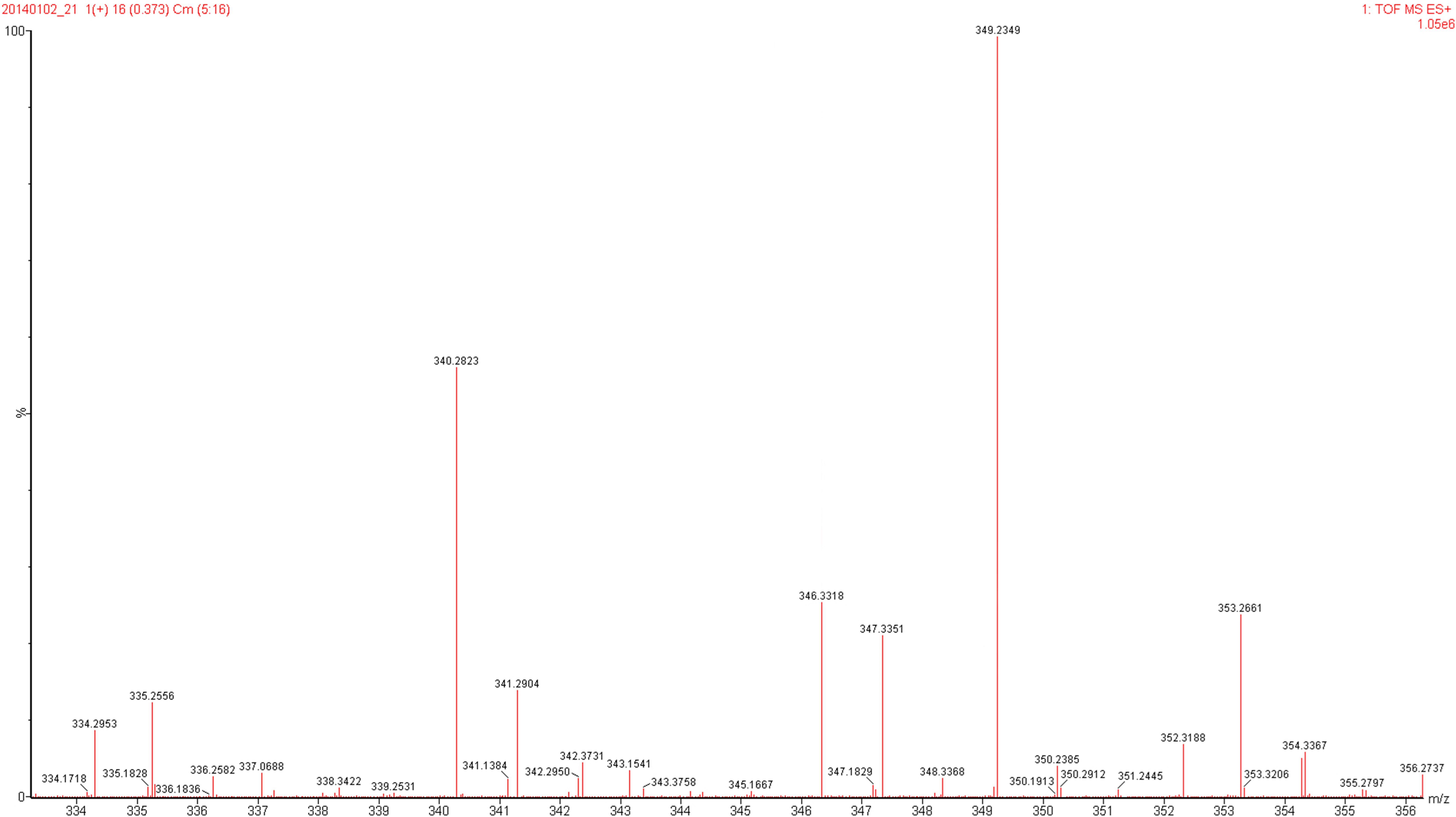

Supplement: S17 Fig — (TIF) [file pone.0116922.s017.tif]

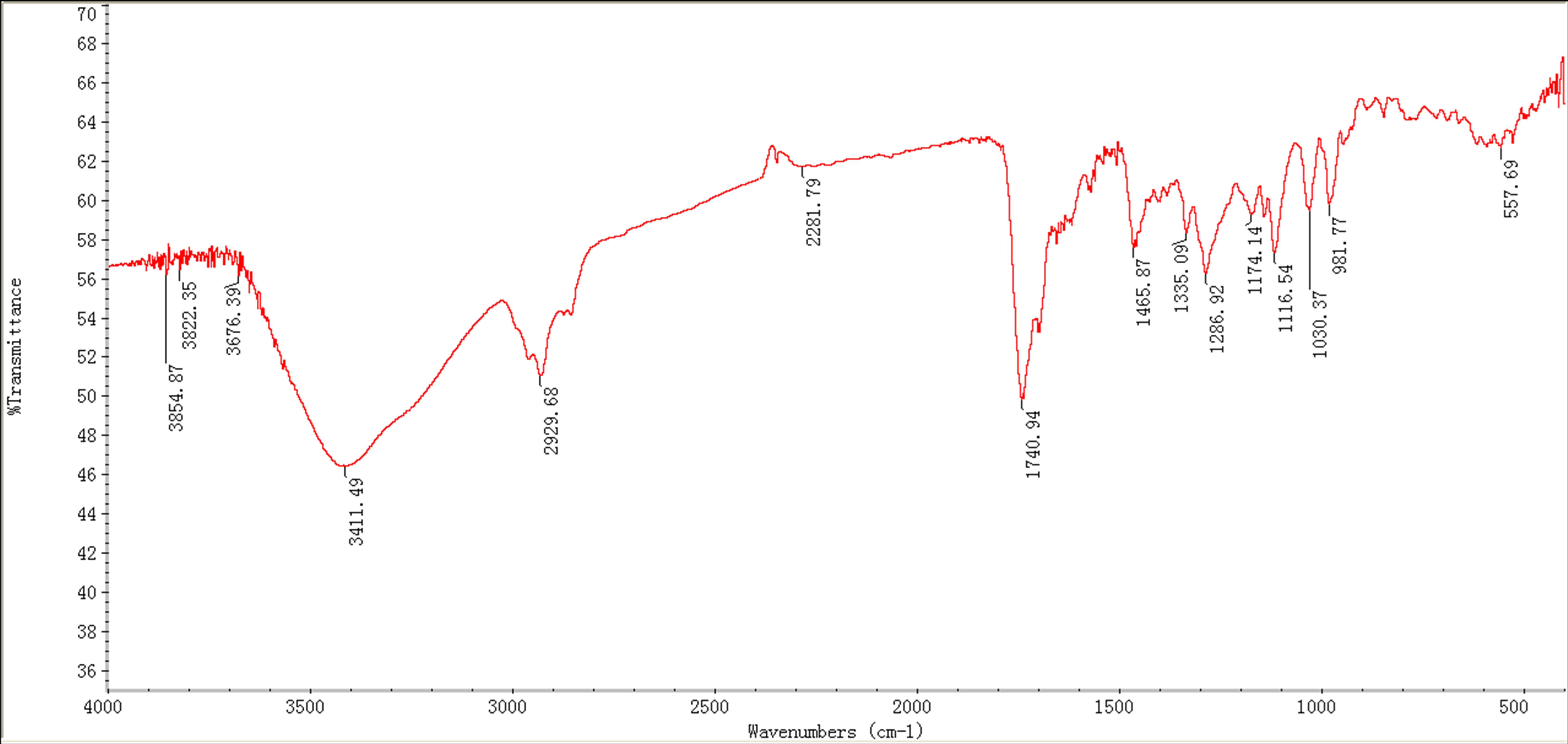

Supplement: S18 Fig — (TIF) [file pone.0116922.s018.tif]

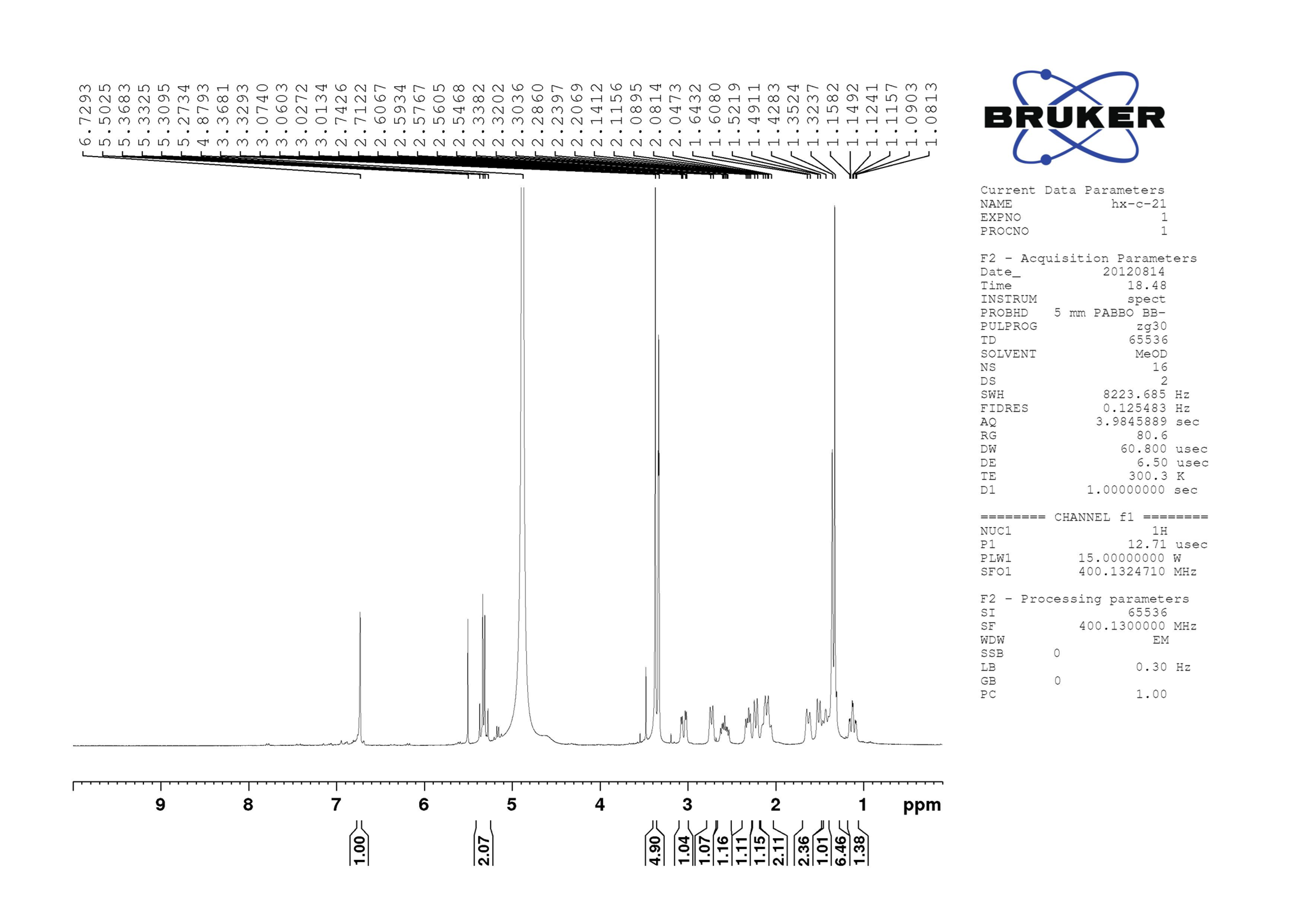

Supplement: S19 Fig — (TIF) [file pone.0116922.s019.tif]

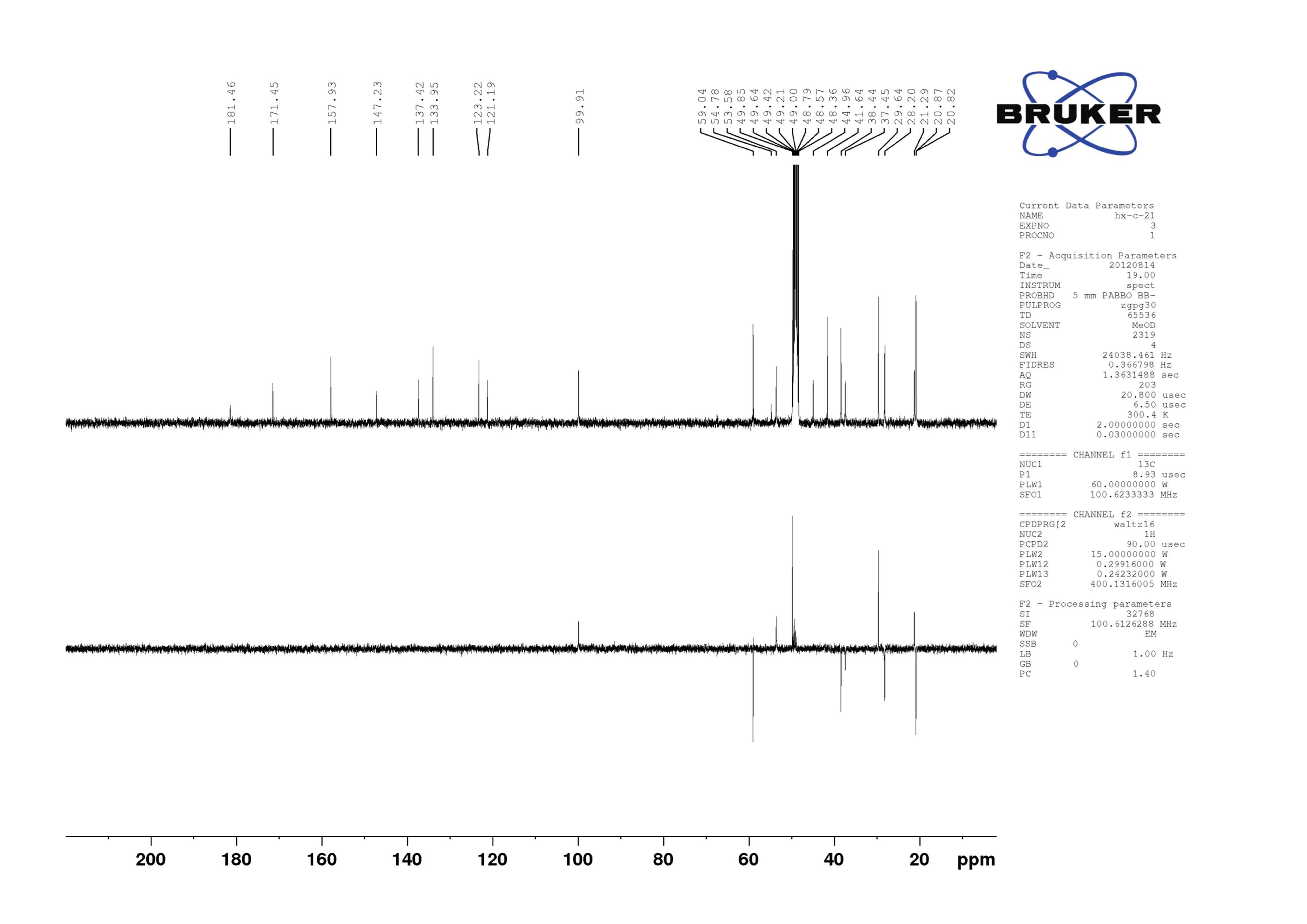

Supplement: S20 Fig — (TIF) [file pone.0116922.s020.tif]

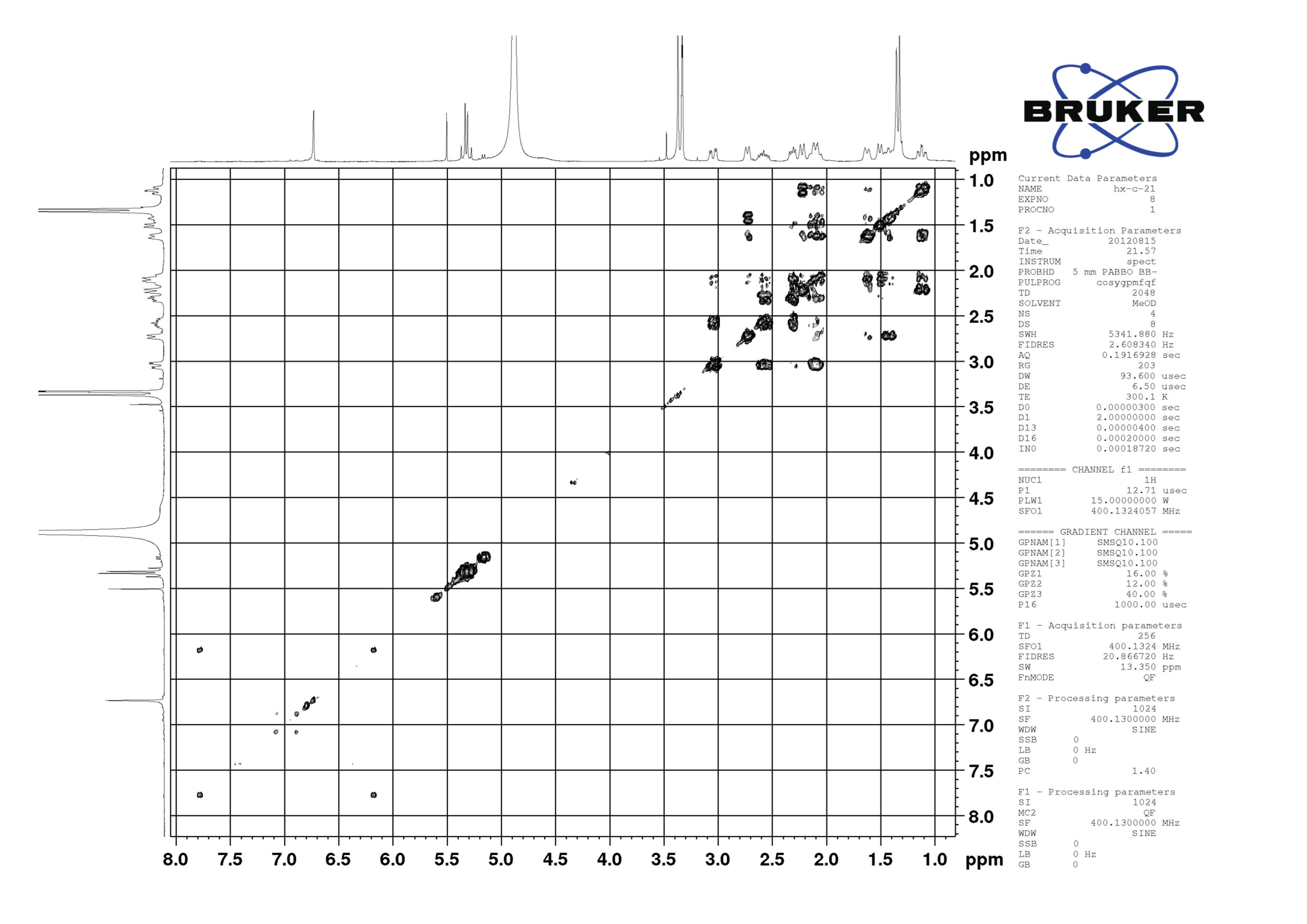

Supplement: S21 Fig — (TIF) [file pone.0116922.s021.tif]

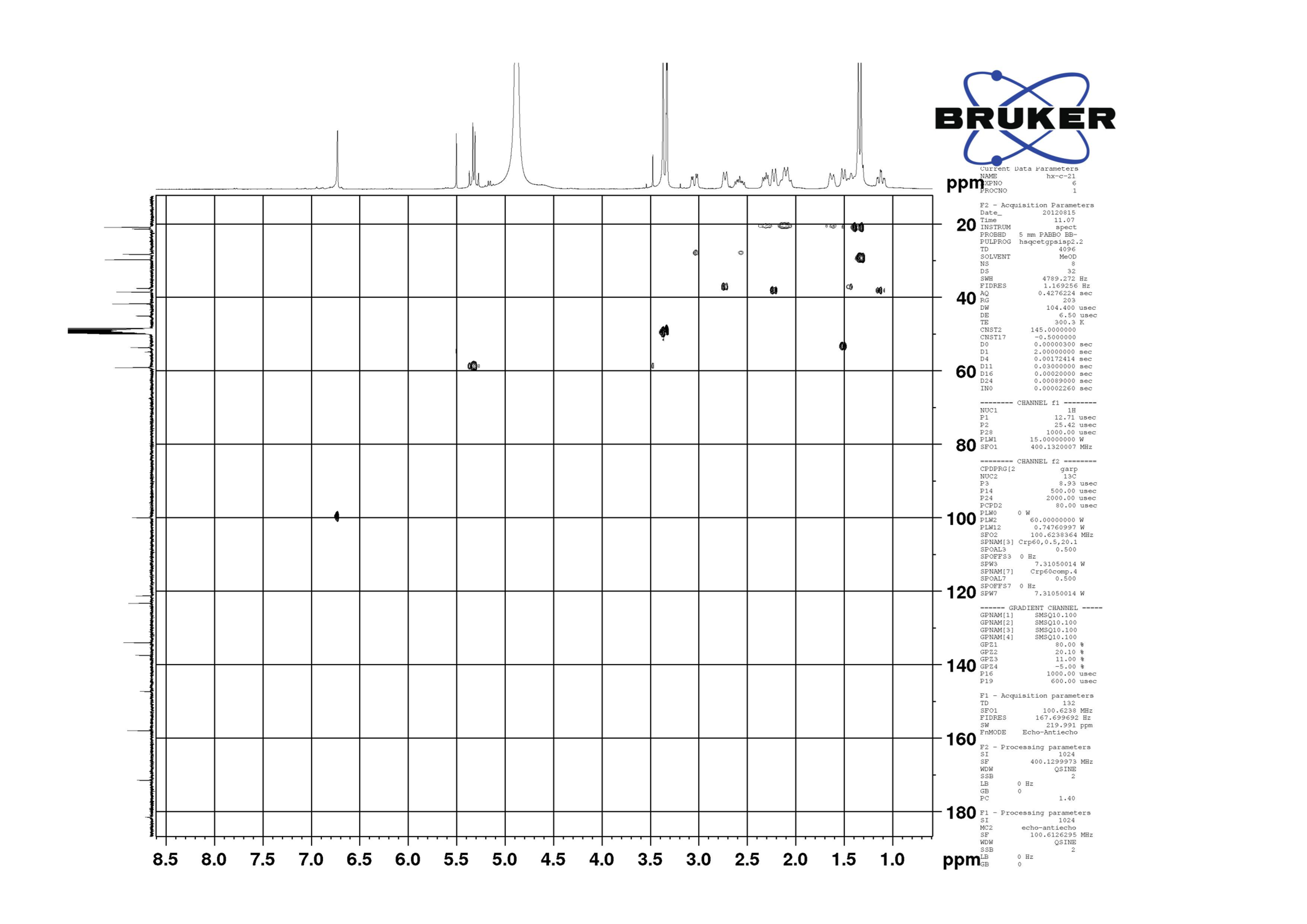

Supplement: S22 Fig — (TIF) [file pone.0116922.s022.tif]

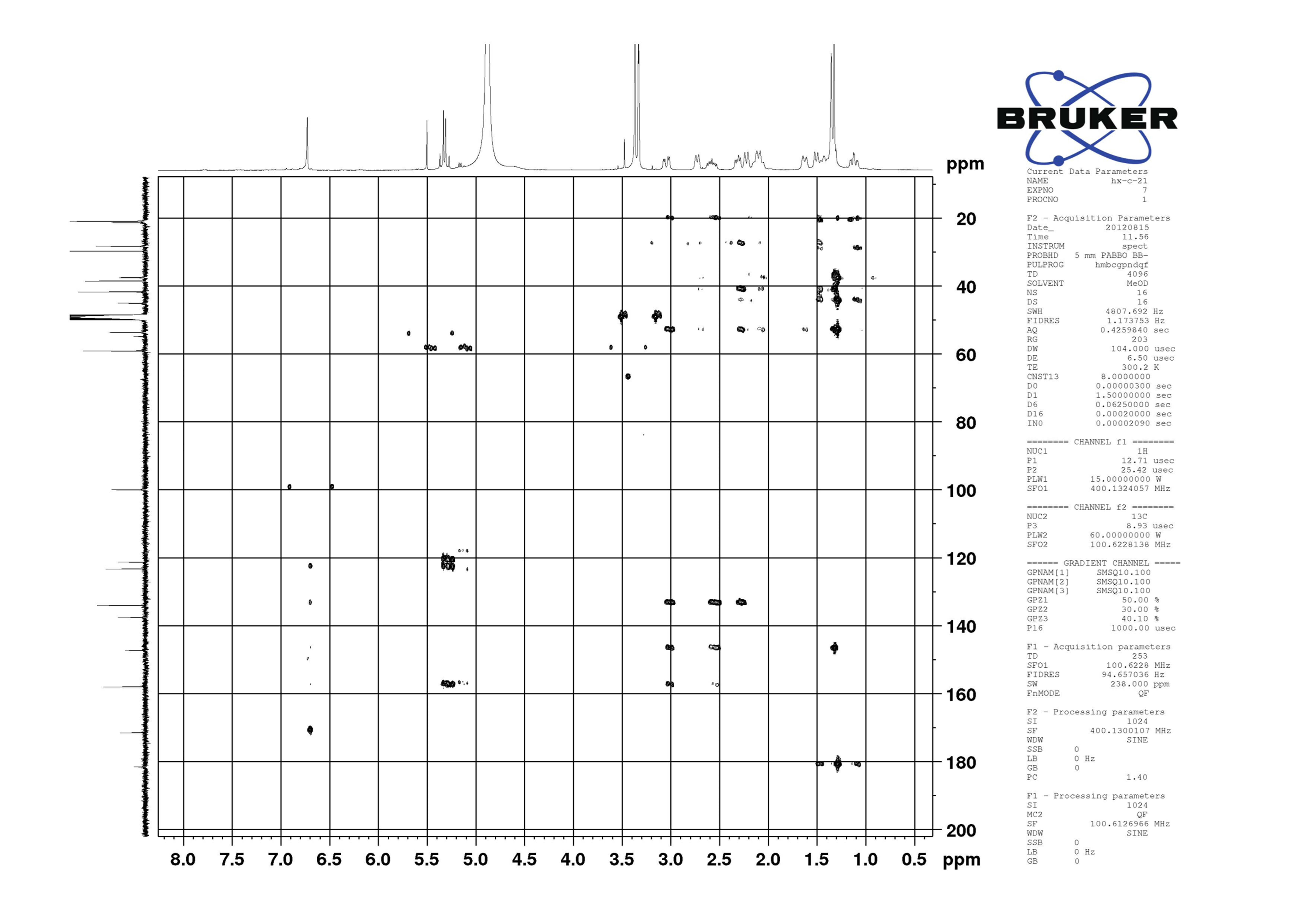

Supplement: S23 Fig — (TIF) [file pone.0116922.s023.tif]

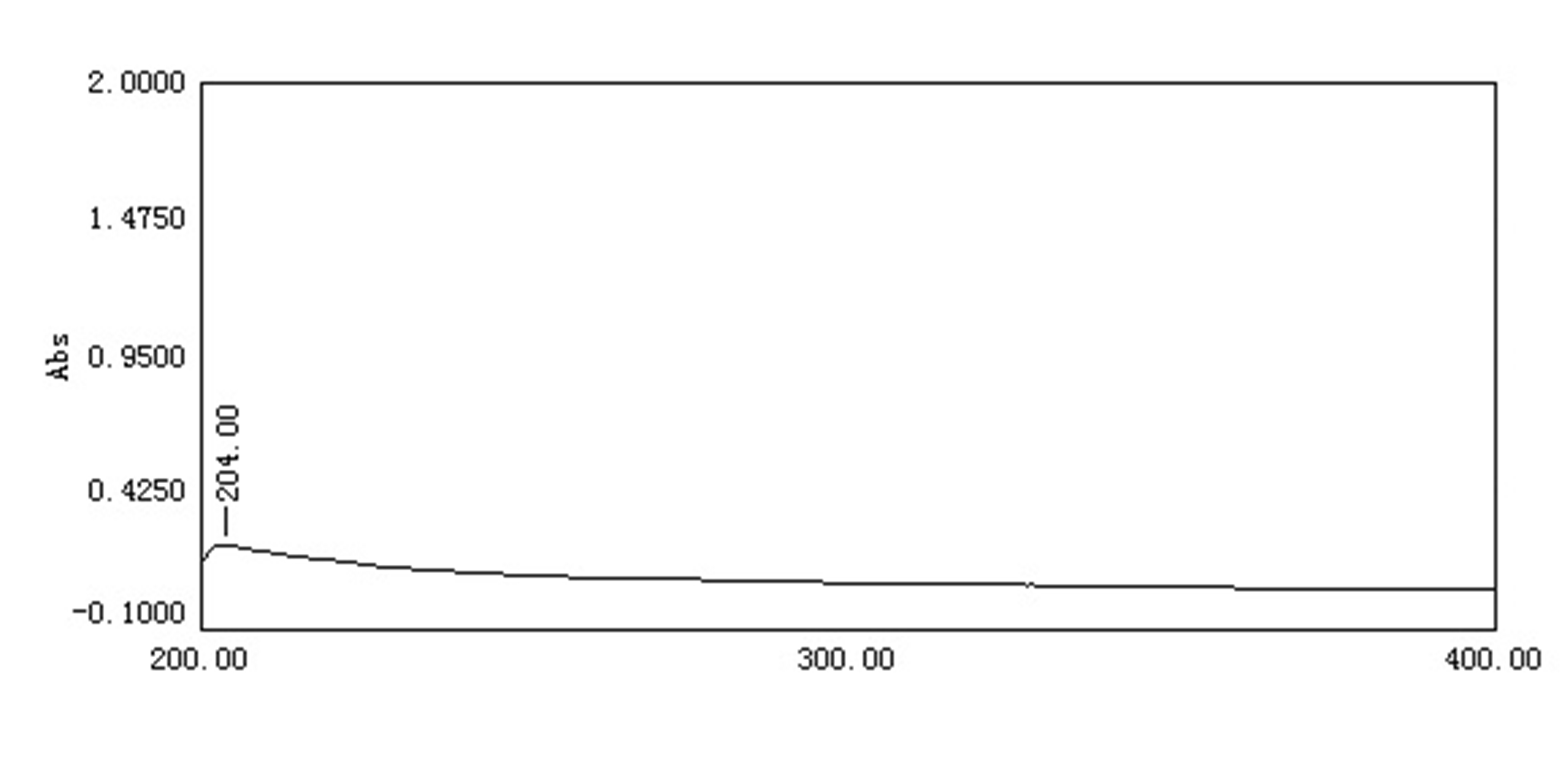

Supplement: S24 Fig — (TIF) [file pone.0116922.s024.tif]

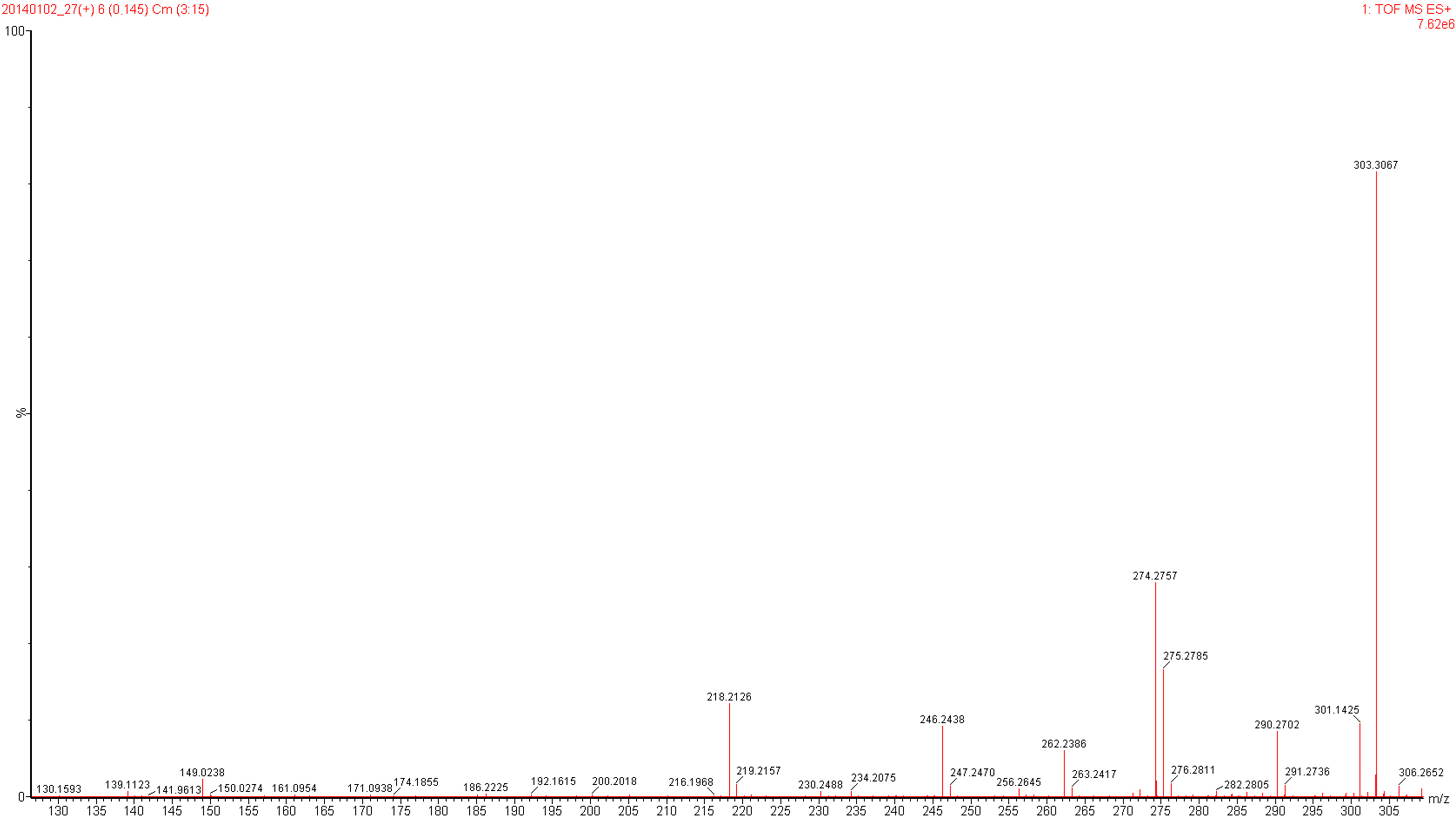

Supplement: S25 Fig — (TIF) [file pone.0116922.s025.tif]

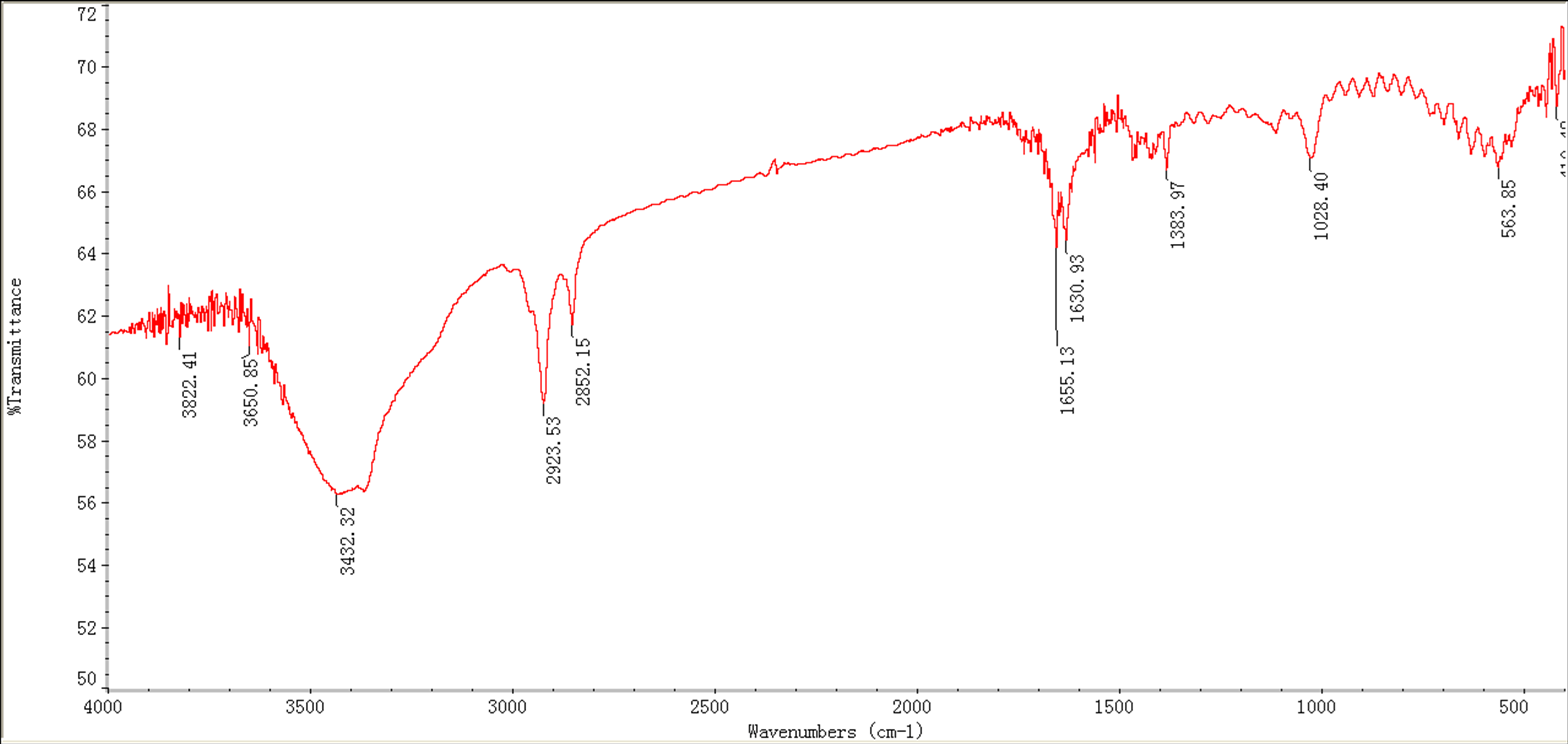

Supplement: S26 Fig — (TIF) [file pone.0116922.s026.tif]

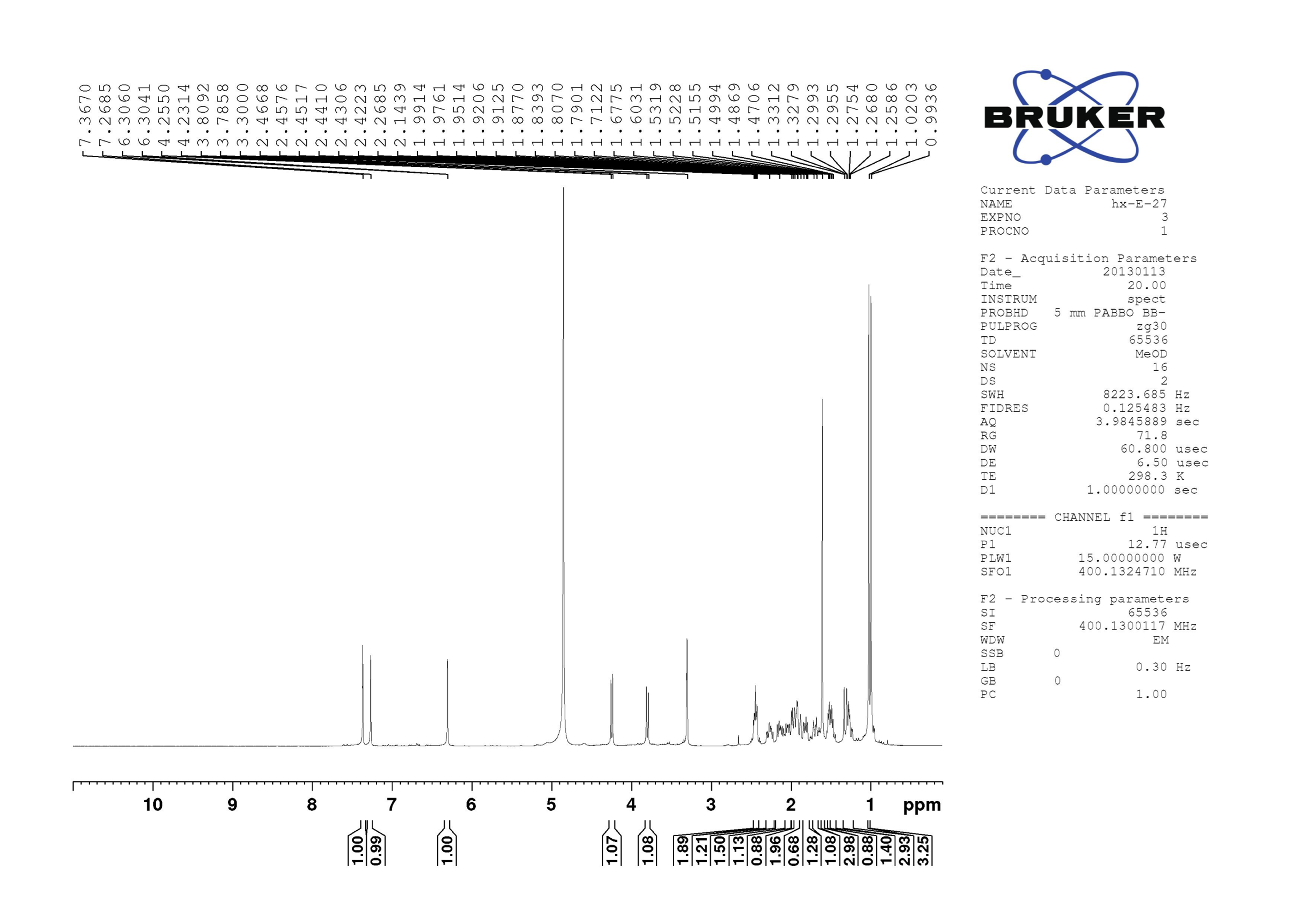

Supplement: S27 Fig — (TIF) [file pone.0116922.s027.tif]

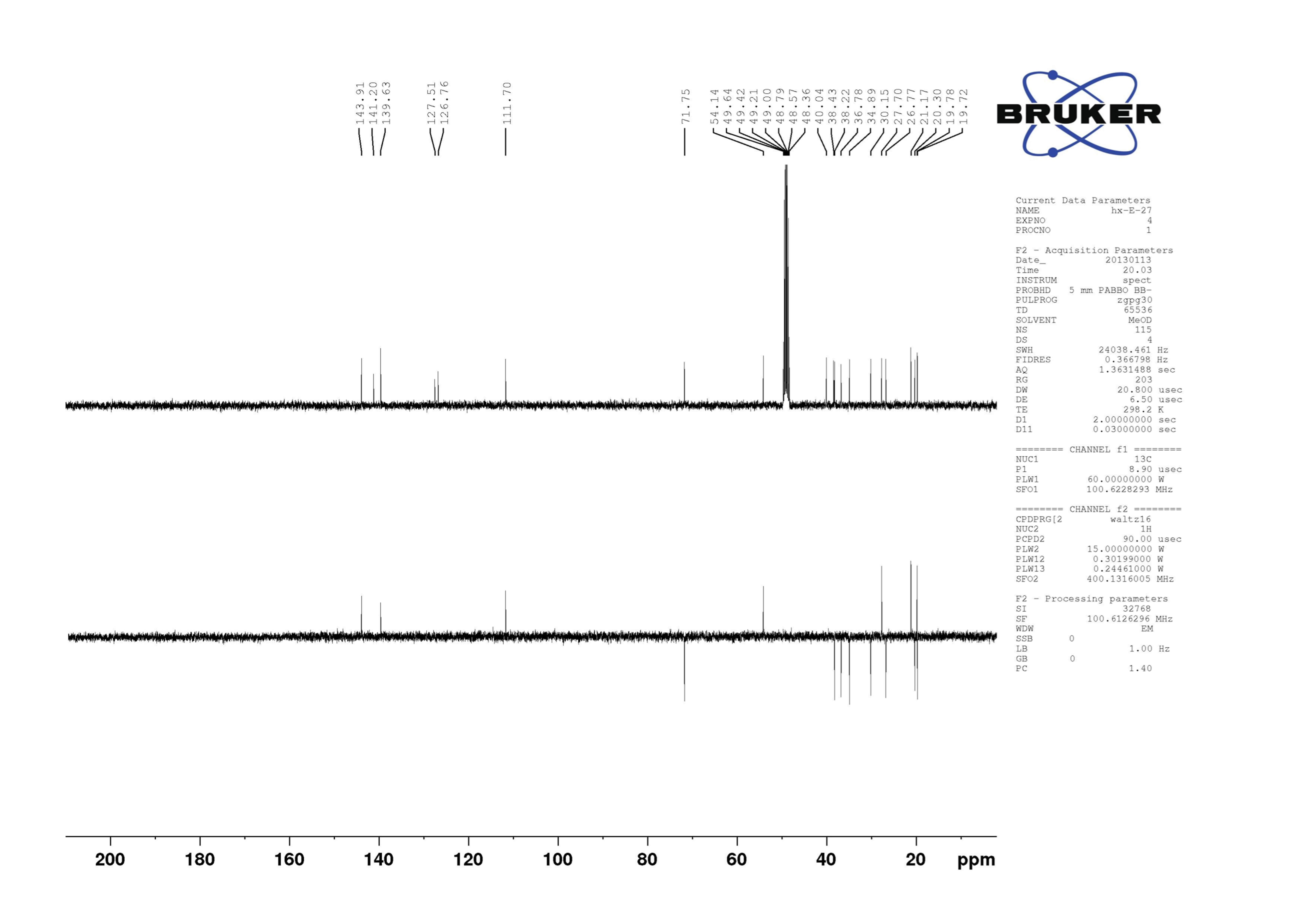

Supplement: S28 Fig — (TIF) [file pone.0116922.s028.tif]

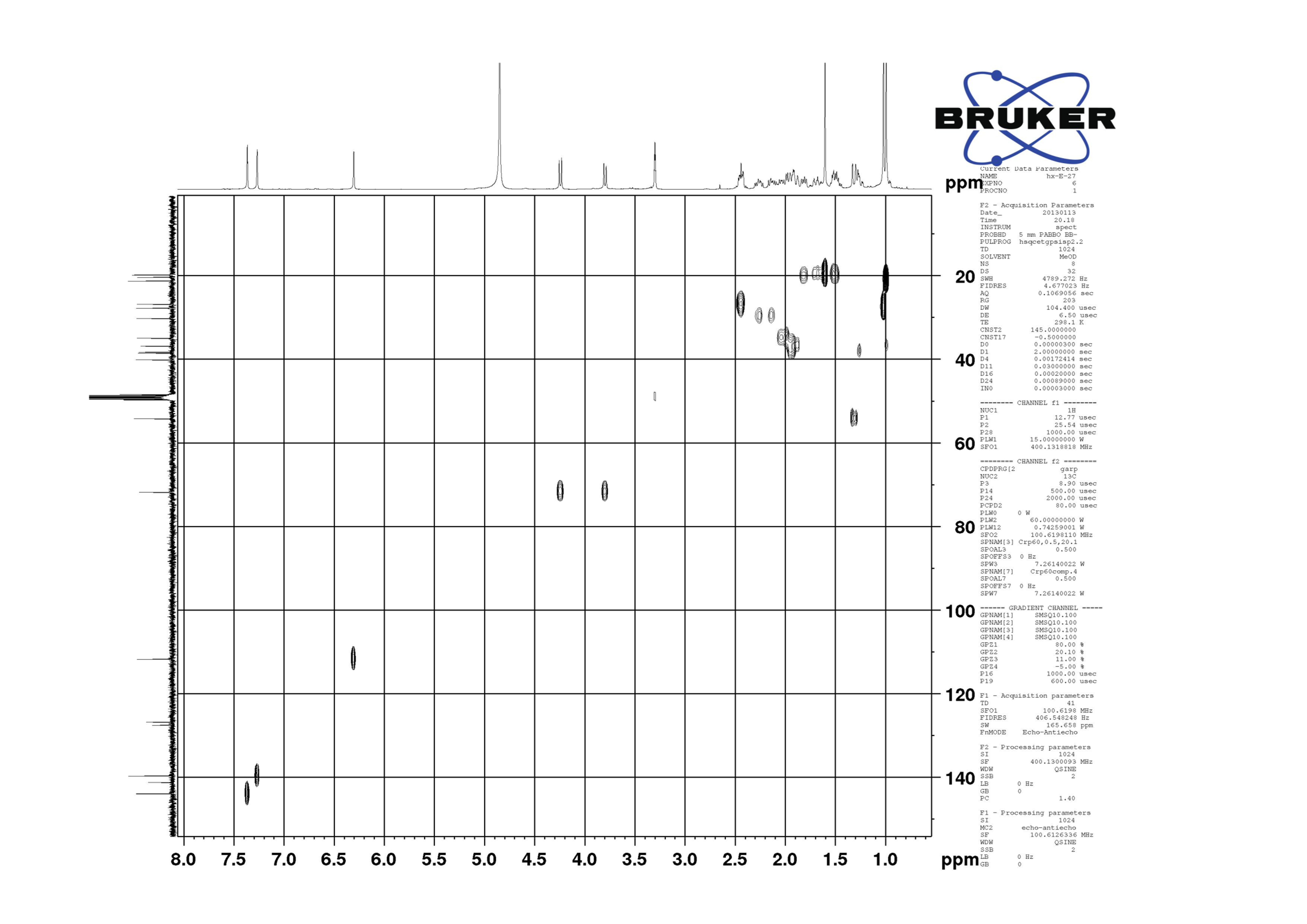

Supplement: S29 Fig — (TIF) [file pone.0116922.s029.tif]

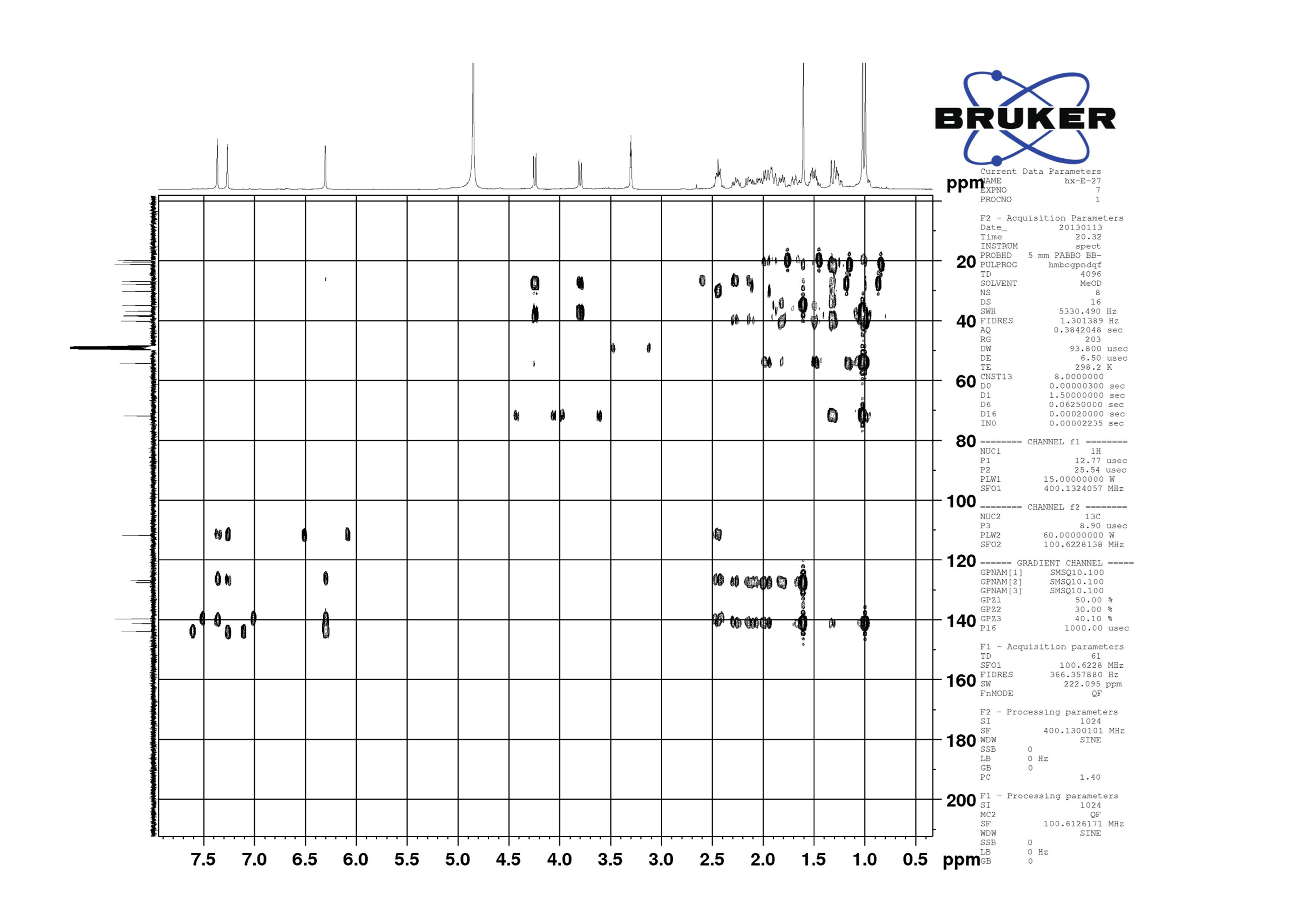

Supplement: S30 Fig — (TIF) [file pone.0116922.s030.tif]

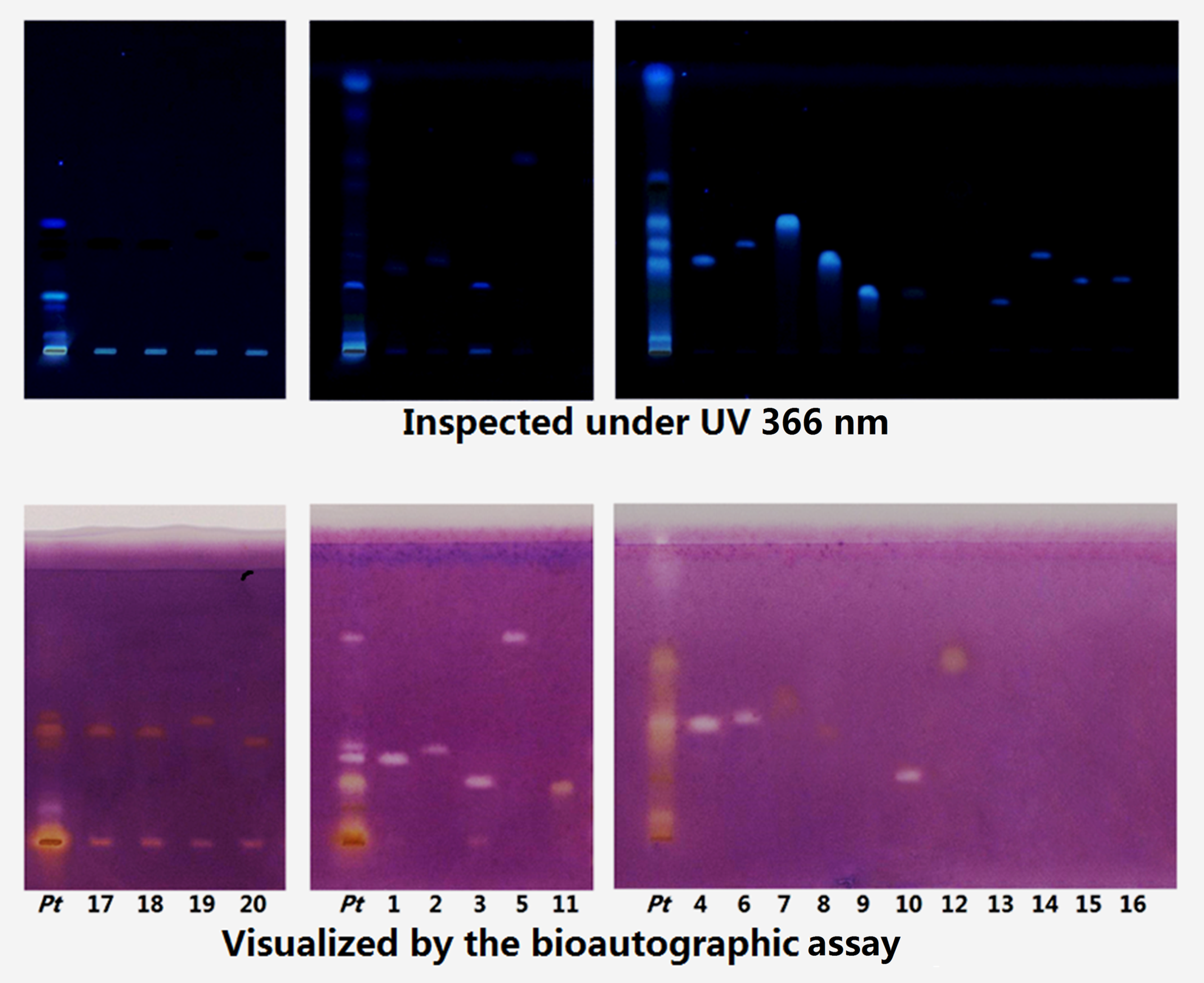

Supplement: S31 Fig — Twenty microliters of the ethyl acetate fraction (Pt, 5 mg/mL) of P. tuberosa and 4 μL of the pure isolates (2.5 mM) were applied as bands on TLC plates. The first plate was eluted with petroleum ether/ethyl acetate (10:1), the second plate was eluted with trichloromethane/methanol (12:1), and the third plate was eluted with ethyl acetate/methanol/water (15:2:1). (TIF) [file pone.0116922.s031.tif]
