# Supplementary material for: Rapid Identification of α-Glucosidase Inhibitors from Phlomis tuberosa by Sepbox Chromatography and Thin-Layer Chromatography Bioautography
Source: PLoS One. 2015 Feb 6;10(2):e0116922. doi: 10.1371/journal.pone.0116922 (PMC4319760; doi:10.1371/journal.pone.0116922)
Supplement: S1 Table — (DOC) [file pone.0116922.s032.doc]

**Table S1. The fractionation conditions of the ethyl acetate extract of *Phlomis. tuberosa* using the Sepbox system**

| 1^st^Separation |  | Elution time | water/Acetonitril | Results |
| --- | --- | --- | --- | --- |
| C4 RP HPLC column  32×125 mm  10μm, 40 mL/min |  | 0-10 min  10-50 min  50-65 min | 100/0  100/0-0/100  0/100 | 15 SPE-Trap |
| 2^st^Separation |  | Elution time | water/Acetonitril/Methanol | Results^e^ |
| Step 1 | SPE2 (T^a^ 17-20 min)  Column 2.4^b^ | 0-60min  60-65 | 100/00/00-90/10/00  00/50/50-00/50/50 | No peak |
| Step 2 | SPE3 (T 17-20 min)  Column 2.4 | 0-60min  60-72min | 95/00/05-80/00/20  00/50/50-00/50/50 | No peak |
| Step 3 | SPE4 (T 17-20 min)  Column 2.4 | 0-60min  60-72min | 98/00/02-85/00/15  00/50/50-00/50/50 | No peak |
| Step 4 | SPE5 (T 17-20 min)  Column 2.4 | 0-60min  60-72min | 95/00/05-80/00/20  00/50/50-00/50/50 | Pt 1 to 3 |
| Step 5 | SPE6 (T 17-20 min)  Column 2.4 | 0-60min  60-72min | 95/00/05-80/00/20  00/50/50-00/50/50 | Pt 4 to 9 |
| Step 6 | SPE8 (T 17-20 min)  Column 2.5^c^ | 0-60min  60-72min | 95/00/05-80/00/20  00/50/50-00/50/50 | Pt 10 to 17 |
| Step 7 | SPE9 (T 17-20 min)  Column 2.5 | 0-60min  60-72min | 95/00/05-75/00/25  00/50/50-00/50/50 | Pt 18 to 29 |
| Step 8 | SPE10(T 17-20 min)  Column 2.5 | 0-60min  60-72min | 90/00/10-70/00/30  00/50/50-00/50/50 | Pt 30 to 38 |
| Step 9 | SPE11(T 17-20 min)  Column 2.5 | 0-60min  60-72min | 90/00/10-65/00/35  00/50/50-00/50/50 | Pt 39 to 66 |
| Step 10 | SPE12(T 17-20 min)  Column 2.5 | 0-60min  60-72min | 85/00/15-60/00/40  00/50/50-00/50/50 | Pt 67 to 89 |
| Step 11 | SPE14 (T 17-20 min)  Column 2.6^d^ | 0-60min  60-72min | 80/00/20-40/00/60  00/50/50-00/50/50 | Pt 90 to 113 |
| Step 12 | SPE15 (T 17-20 min)  Column 2.6 | 0-50min  50-72min | 75/00/25-00/20/80  00/20/80-00/00/00 | Pt 114 to 130 |
| Step 13 | SPE16 (T 17-20 min)  Column 2.6 | 0-30min  30-72min | 35/20/45-00/20/80  00/20/80-00/50/50 | Pt 131 to 137 |
| Step 14 | SPE17 (T 17-20 min)  Column 2.6 | 0-30min  30-72min | 10/20/70-00/20/80  00/20/80-00/20/80 | Pt 137 to 142 |
| Step 15 | SPE18 (T 17-20 min)  Column 2.6 | 0-30min  30-72min | 10/20/70-00/20/80  00/20/80-00/50/50 | Pt 142 to 150 |

^a^T: Trapping time for the eluents from 1^st^ separation column, ^b^Column 2.4: C18 RP HPLC column (16×250 mm, 5 μm, 40 mL/min), ^c^Column2.5: C18 RP HPLC column (16×250 mm, 5 μm, 40 mL/min), ^d^Column 2.6: C18 Aqueous RP HPLC column (16×150 mm, 5 μm, 40 mL/min),^e^The eluents coming from 2^nd^ separation steps were collected in 150 deep well by 50 mL using an automatic fraction collector through monitoring HPLC peaks from UV (254) and ELSD detectors.
