# Supplementary material for: Rapid Identification of α-Glucosidase Inhibitors from Phlomis tuberosa by Sepbox Chromatography and Thin-Layer Chromatography Bioautography
Source: PLoS One. 2015 Feb 6;10(2):e0116922. doi: 10.1371/journal.pone.0116922 (PMC4319760; doi:10.1371/journal.pone.0116922)
Supplement: S2 Table — (DOC) [file pone.0116922.s033.doc]

**Table S2.** Solvent gradient program of HPLC analysis

| Time (min) | Water (A, %) | Acetonitril (B, %) |
| --- | --- | --- |
| 0 | 95 | 5 |
| 8 | 85 | 15 |
| 13 | 80 | 20 |
| 23 | 80 | 20 |
| 25 | 60 | 40 |
| 40 | 50 | 50 |
| 60 | 0 | 100 |
| 70 | 0 | 100 |
